# Vascular Plant Flora of the Koktau Mountains (Kalba Ridge): Diversity, Phytogeographical Relationships, and Conservation Significance in the Kazakhstan Altai

**DOI:** 10.3390/biology15151309

**Published:** 2026-08-05

**Authors:** Aidar Sumbembayev, Yuriy Kotukhov, Alevtina Danilova, Sławomir Nowak, Yuliya Genievskaya

**Affiliations:** 1 Altai Botanical Garden, Ridder 070000, Kazakhstan; kotukhov35@bk.ru (Y.K.); a-n-danilova@yandex.ru (A.D.); 2 Department of Plant Taxonomy and Nature Conservation, Faculty of Biology, University of Gdansk, 80-308 Gdansk, Poland; slawomir.nowak@ug.edu.pl; 3 Institute of Plant Biology and Biotechnology, Almaty 050040, Kazakhstan; julia.genievskaya@gmail.com

**Keywords:** endemic species, floristic diversity, mountain flora, phytogeography, species richness

## Abstract

The Koktau Mountains, located within the Kalba Highlands of Eastern Kazakhstan, represent an important yet insufficiently studied center of plant diversity in the Kazakhstan Altai. Field surveys conducted between 2018 and 2025 yielded more than 2700 herbarium specimens and were complemented by a revision of collections deposited in the ABG, AA, LE, and MW herbaria. Taxonomic, ecological, and incidence-based comparative analyses were used to characterize the flora and evaluate its relationships with neighboring regions. We recorded 817 accepted vascular plant taxa at species and infraspecific ranks, belonging to 356 genera and 79 families. The flora combines boreal, Mediterranean, and Turanian elements, reflecting the transitional position of the Koktau Mountains between the Altai mountain systems and the steppes of Eastern Kazakhstan. Twenty-seven rare and threatened taxa were identified, including 16 species listed in the Red Data Book of Kazakhstan and two species endemic to Kazakhstan, one of which, *Elymus sibinicus*, is currently known only from the Koktau Mountains. Floristic similarity was greatest with the Bukhtarma Mountains and Ulba Range. Because the compared inventories differ in area and survey history, these incidence-based relationships are interpreted descriptively. Together with the low number of endemic taxa, these relationships are consistent with the interpretation of the Koktau Mountains as a transitional flora influenced by regional floristic exchange.

## Simple Summary

The Koktau Mountains in eastern Kazakhstan are an important but insufficiently studied center of plant diversity. This study aimed to document the plant species of the region and evaluate their importance for biodiversity conservation. Based on field surveys conducted between 2018 and 2025 and the examination of herbarium collections, we recorded 817 vascular plant taxa within an area of approximately 510 km^2^. Despite its relatively limited area, the Koktau Mountains support a floristically heterogeneous assemblage associated with forest, meadow, steppe, wetland, and rocky habitats. Comparisons with neighboring floras were interpreted cautiously because the regions differ considerably in area and survey history. We also identified 27 rare and threatened taxa, including 16 species protected at the national level and two species endemic to Kazakhstan. One of these, *Elymus sibinicus*, is currently known only from the Koktau Mountains. Our findings demonstrate that this region is an important refuge for plant diversity and a priority area for conservation, monitoring, and sustainable management of natural resources.

## 1. Introduction

Mountain ecosystems are major centers of global biological diversity, although their floristic richness and endemism vary considerably among mountain systems according to latitude, area, topographic heterogeneity, climatic history, and geographical isolation [1,2]. Their complex environmental gradients and geological histories create opportunities for habitat specialization, the coexistence of taxa from different regional species pools, and, in sufficiently isolated systems, evolutionary diversification, frequently resulting in high species richness and endemism. The Altai Mountain Country, located at the junction of Central and Northern Asia, is recognized as one of the major floristic centers of Eurasia and constitutes a key component of the Altai–Sayan Ecoregion [3]. Recent assessments indicate that the Kazakhstan portion of the Altai supports more than 2400 species of vascular plants [4]. Furthermore, the region contains numerous centers of plant speciation and refugial habitats that have contributed to the preservation of floristic diversity since the Late Pleistocene [5].

Mountain floras are assembled through the interaction of regional species pools, environmental filtering along climatic and topographic gradients, dispersal among neighboring landscapes, and evolutionary differentiation promoted by geographical isolation [6]. Consequently, high floristic richness may result either from local diversification or from the overlap of species belonging to different biogeographical elements [7]. These alternatives can be evaluated by combining taxonomic and ecological spectra with comparisons of floristic composition, patterns of regional similarity, and regional endemism [8]. Floristic inventories therefore provide not only baseline documentation but also a basis for examining regional connectivity, ecological heterogeneity, and conservation value.

**Figure 1:**
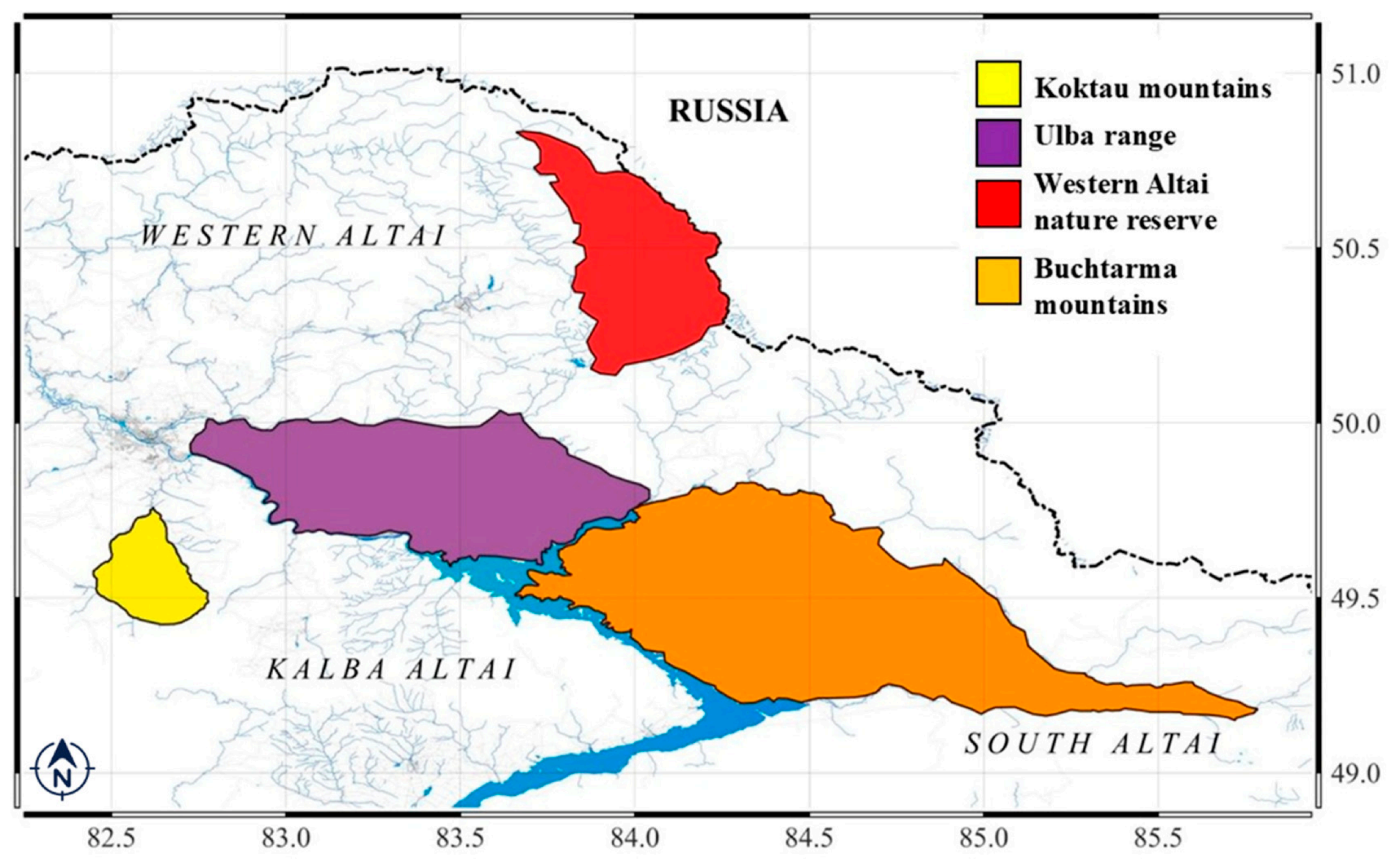
Location of the Koktau Mountains and the three regional floras used for comparison within the Kazakhstan Altai: Ulba Range, West Altai Nature Reserve, and Bukhtarma Mountains. Map axes show geographical longitude and latitude in decimal degrees.

The exceptional floristic richness of the Kazakhstan Altai is determined by its unique geographical position at the intersection of several floristic provinces, its highly dissected mountain relief, and the diversity of climatic, geological, and edaphic conditions. The territory encompasses a broad range of ecosystems, extending from dry steppes and forest-steppes to taiga forests, alpine meadows, subalpine shrublands, and high-mountain tundra communities [9]. Such environmental diversity promotes the coexistence of species belonging to different phytogeographical elements and contributes to the formation of highly heterogeneous plant communities. Consequently, the Kazakhstan Altai supports high vascular-plant richness and a broad range of mountain habitats within Kazakhstan [4,9] (Figure 1).

Within the Kazakhstan Altai, the Kalba Ridge occupies a distinctive biogeographical position. Although lower in elevation than the major ranges of the Western and Southern Altai, it connects these mountain systems with the steppe regions of Eastern Kazakhstan and functions as an important corridor for species dispersal. This setting contributes substantially to the landscape-scale biodiversity of the region.

Within this regional setting, the Koktau Mountains were expected to differ from the neighboring mountain-taiga floras because their moderate elevation, granitic relief, Sibin wetland system, and proximity to the steppes of Eastern Kazakhstan create a particularly heterogeneous habitat mosaic. The juxtaposition of forest, meadow, wetland, rocky, and dry-steppe habitats may promote the overlap of boreal, montane, and southern floristic elements, while the comparatively weak geographical isolation is expected to limit local endemism.

The Koktau Mountains are situated within the East Kazakhstan Region and belong to the Kalba Highlands [10,11]. They are located in the central forested part of the eastern Kalba Ridge. The central portion of the Koktau Mountains contains the Sibin Depression, characterized by a system of small mountain rivers and lakes that significantly influence local ecological conditions and vegetation patterns [12].

The relief of the Koktau Mountains is complex and highly heterogeneous. Extensive areas are occupied by strongly dissected intrusive massifs composed predominantly of granitic rocks. One of the characteristic geomorphological features of the region is the presence of numerous concave intermountain depressions that form distinct ecological habitats [13]. The soils of the Koktau Mountains follow the patterns of both latitudinal and altitudinal zonality and are represented mainly by dark chestnut soils typical of the steppe zone.

Climatic conditions in the study area are characterized by pronounced continentality. The period of active plant vegetation generally lasts between 100 and 120 days [14]. Despite the relatively short growing season, the region supports a diverse vegetation cover formed by the interaction of mountain, forest, steppe, and meadow ecosystems. Unlike many mountain systems, the vegetation of the Koktau Mountains exhibits only weak expression of classical altitudinal zonation. Instead, the vegetation mosaic is composed primarily of forest, steppe, and meadow communities, supplemented by fragmented aquatic and riparian vegetation associated with lakes and river valleys. The relatively weak expression of classical altitudinal zonation is primarily associated with the moderate elevation range and comparatively low relief amplitude of the Koktau Mountains. Under the strongly continental regional climate, slope exposure, local moisture availability, river valleys, and intermountain depressions therefore exert a greater influence on vegetation distribution than elevation alone [15].

The distribution of woody vegetation is closely linked to local topography and moisture availability. Floodplains and river valleys are occupied by communities dominated by *Populus laurifolia*, *Betula pendula*, and *Salix alba* [16], while deep shaded ravines, gullies, and north-facing slopes additionally support *Populus tremula* and *Abies sibirica* [17]. Pine forests represented by *Pinus sylvestris* occur mainly on rocky slopes and ridge summits [18]. Nevertheless, forest vegetation remains relatively fragmented, and both coniferous and deciduous forests occupy limited areas within the overall landscape structure.

The study of Kazakhstan’s flora has acquired particular importance in the context of ongoing global biodiversity decline and accelerating environmental change. Contemporary floristic investigations in Kazakhstan are directed toward documenting actual species diversity, clarifying the distribution of taxa, identifying conservation priorities, and monitoring vegetation dynamics. Numerous studies have focused on floristic inventories of individual regions and protected areas [19,20,21,22,23,24,25,26,27], assessments of the current status of economically important and valuable plant species [28,29,30,31,32], investigations of rare and endangered taxa [33,34,35,36,37], and the discovery of new records for the flora of Kazakhstan [38,39,40,41].

Considerable progress has also been achieved in taxonomic and floristic research through the compilation of checklists of rare and endemic plants [42,43,44], revisions of individual sections and genera [42,43,44,45,46,47,48,49,50,51,52,53], and comprehensive treatments of entire plant families [54,55]. These studies have substantially improved our understanding of the floristic composition and taxonomic diversity of Kazakhstan.

Particularly intensive floristic research has been conducted within the Kazakhstan Altai, one of the richest botanical regions of the country [56,57]. During recent decades, numerous floristic novelties have been reported from Eastern Kazakhstan, including discoveries and new distribution records within the genera *Fritillaria* [58,59], *Geranium* [58], *Pedicularis* [59], *Potentilla* [60], *Astragalus* [61], and *Sanguisorba* [62]. Floristic inventories have been compiled for several territories of Eastern Kazakhstan [63,64], while the population status of rare and threatened species belonging to genera such as *Rhodiola* [65,66,67], *Tulipa* [68], and *Prunus* [69] has been investigated in detail.

Despite these achievements, floristic knowledge of many parts of the Kazakhstan Altai remains incomplete. Existing studies are frequently restricted to individual taxa, specific localities, or particular categories of plants. Consequently, substantial gaps remain in our understanding of regional floristic diversity, especially in territories that have received limited attention from botanists. The Koktau Mountains represent one such area. A previous regional study documented 576 vascular plant species from the southwestern and northeastern parts of the Koktau Mountains and provided a preliminary analysis of their taxonomic and ecological structure [70]. However, that investigation did not encompass the entire mountain system, did not integrate the extensive herbarium material examined in the present study, and did not evaluate floristic relationships with neighboring phytogeographical regions or conservation statuses across multiple national and international sources. The present study therefore extends the earlier inventory through seven years of field surveys covering the principal habitats of the Koktau Mountains, revision of collections from four herbaria, updated nomenclature, and comparative floristic analyses. The objective was to determine the taxonomic, ecological, phytogeographical, and conservation characteristics that define the position of the Koktau flora within the Kazakhstan Altai. Specifically, the study addressed the following questions: (1) What taxonomic, ecological, and growth-form patterns characterize the vascular flora of the Koktau Mountains? (2) Which neighboring regional floras show the greatest compositional affinity with the Koktau flora? (3) Does the flora primarily reflect regional floristic exchange and overlap or pronounced local differentiation? (4) Which endemic, rare, and nationally protected taxa and habitats should be prioritized for conservation and monitoring? The study is therefore an incidence-based floristic inventory and regional checklist comparison rather than a quantitative analysis of community abundance, habitat-level diversity, or species occupancy.

## 2. Materials and Methods

### 2.1. Study Area

**Figure 2:**
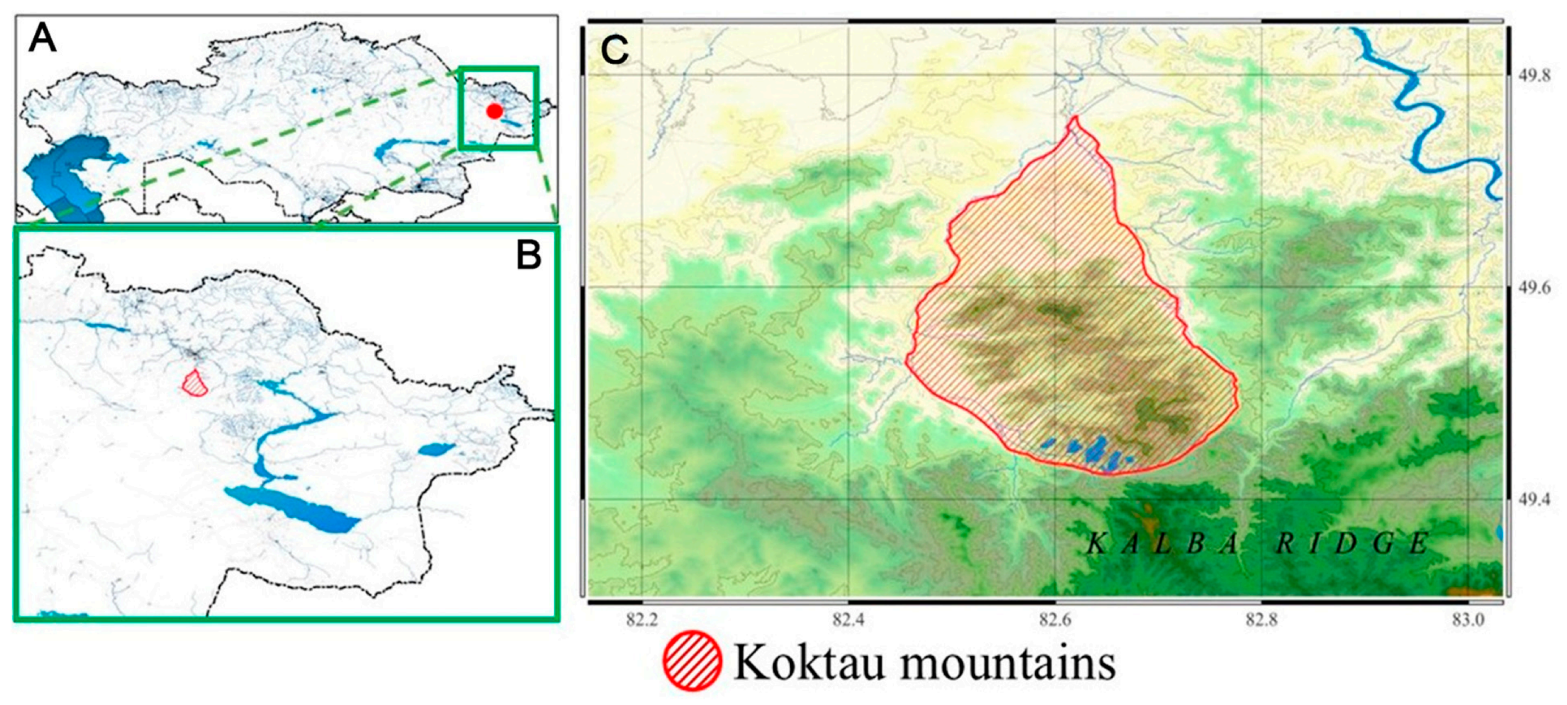
Location of the study area: (**A**) position of East Kazakhstan within Kazakhstan; (**B**) position of the Koktau Mountains within East Kazakhstan and the Kalba Ridge; and (**C**) boundary of the approximately 510 km^2^ surveyed territory. Coordinates are shown in decimal degrees.

The Koktau Mountains are located approximately between 49.4–49.8° N and 82.4–82.8° E, and occupy an area of approximately 510 km^2^ (Figure 2). The surveyed territory extends from approximately 505 to 1455 m a.s.l. The mountain system is situated in the central forested part of the eastern Kalba Ridge and represents a transitional territory between the mountain ecosystems of the Kazakhstan Altai and the steppe landscapes of Eastern Kazakhstan.

### 2.2. Field Surveys and Herbarium Studies

The present checklist is based primarily on original field collections conducted by the authors during botanical expeditions between 2018 and 2025. Field investigations covered the entire territory of the Koktau Mountains and all major vegetation types. Field routes were planned following preliminary reconnaissance [71] and available topographic information to cover the principal geomorphological units and vegetation types of the Koktau Mountains. These included forest and shrub communities, meadow and steppe habitats, river valleys, lakeshores and wetlands, intermountain depressions, granitic outcrops, rocky slopes, ravines, and ridge summits. The routes were distributed throughout the study area and repeatedly surveyed in different years and at different stages of the vegetation season to increase the probability of recording both early-flowering and late-season taxa. The study followed a floristic inventory design rather than a standardized plot- or transect-based abundance survey; therefore, sampling effort was not expressed as a fixed number of plots per unit area. Habitat polygons and the proportion of each habitat covered by the survey were not quantified; consequently, the resulting data support an incidence-based floristic inventory but not habitat-area-standardized estimates of species richness or occupancy. Areas with difficult rocky terrain may nevertheless have been sampled less intensively than accessible valleys and slopes.

In total, the surveys comprised 213 field days. Coordinates were recorded using a Garmin eTrex 32x handheld receiver (Garmin International, Inc., Olathe, KS, USA), with a positional accuracy of 3–5 m under open-field conditions. Voucher specimens were collected for all recorded taxa whenever possible. In total, more than 2700 herbarium specimens were collected and processed during the study period. Duplicate sheets of the same collection event were not counted separately in the reported total. Records with ambiguous locality information or uncertain identification were excluded.

In addition to field collections, herbarium specimens deposited in the Herbarium of the Altai Botanical Garden (ABG, Ridder, Kazakhstan), the Herbarium of the Institute of Botany and Phytointroduction (AA, Almaty, Kazakhstan), the Herbarium of the Komarov Botanical Institute (LE, Saint Petersburg, Russia), and the Herbarium of the Main Botanical Garden (MW, Moscow, Russia) were examined. Herbarium data were used to verify species occurrences, clarify taxonomic identifications, and supplement field observations.

### 2.3. Taxonomic Treatment and Ecological Assessment

Species identification was carried out using the principal floristic treatments for the region, including Flora of Kazakhstan [72], Flora of the USSR [73], and Flora of Siberia [74], as well as by comparison with authenticated herbarium specimens. Scientific names and nomenclature follow Plants of the World Online (POWO) [75] and synonymous names were consolidated. Family circumscription follows the Angiosperm Phylogeny Group classification system APG IV [76], lycophytes and ferns follow the Pteridophyte Phylogeny Group classification (PPG I) [77], and gymnosperm families follow Christenhusz et al. [78]. Under PPG I, horsetails are included within the fern class Polypodiopsida as subclass Equisetidae rather than treated as a separate major division. Within families, genera and species are arranged alphabetically. Taxonomic author citations are provided in the formal checklist (Table S1) and are omitted from the running text, figure captions, and analytical tables.

Ecological ecomorphs were assigned according to the moisture conditions and substrate preferences observed at the collection localities and described in regional floristic treatments and herbarium labels. Field observations were used as the primary source, while published habitat descriptions were used to verify assignments. Because many species occupy transitional conditions, intermediate categories were retained. The following operational categories were used: hydrophytes (plants growing permanently in aquatic habitats); hygrophytes (plants of water-saturated soils, wetlands, and persistently wet shores); mesohygrophytes (plants of moist to wet habitats with a stronger affinity to saturated conditions); hygromesophytes (plants of moderately moist habitats that tolerate periodic water saturation); mesophytes (plants of moderately moist, generally well-drained habitats); xeromesophytes (plants of moderately dry habitats showing greater affinity to mesic than strongly xeric conditions); mesoxerophytes (plants predominantly associated with dry habitats but less drought-specialized than xerophytes); xerophytes (plants of persistently dry habitats subject to marked water deficit); petrophytes (plants primarily associated with rocks, rocky outcrops, screes, and rock crevices); mesopetrophytes (plants of comparatively moist or shaded rocky habitats); and xeropetrophytes (plants of dry, exposed rocky substrates). Species were assigned to a single category representing the habitat conditions most characteristic of their occurrence in the Koktau Mountains. For taxa occurring in several habitat types, the assigned category represents the conditions most frequently recorded in the Koktau Mountains and should be interpreted as a predominant regional association rather than the full ecological amplitude of the species. Endemic taxa were identified according to Kubentayev et al. [40], whereas the status of rare and endangered species within the Kazakhstan Altai was determined following Sumbembayev et al. [42].

Life forms were assigned using information from the regional floras, POWO, herbarium labels, and field observations. Woody plants were classified as trees, shrubs, or subshrubs according to stem lignification and growth habit. Herbaceous plants were classified as annual, biennial, or perennial according to the number of growing seasons normally required to complete the life cycle. Slash categories indicate documented variation in life-history duration rather than intermediate growth forms: annual/biennial taxa may complete their life cycle in either one or two seasons; annual/perennial taxa have been reported as annual or short-lived perennial under different environmental conditions; and biennial/perennial taxa may persist beyond the second growing season. Each taxon was assigned to one category according to the regional floristic literature and the growth form observed most consistently in the Koktau Mountains.

For species included in the Red Data Book of Kazakhstan [79], the corresponding national conservation category was recorded. Additionally, conservation assessments from the IUCN Red List [80], the Red Data Book of Altai Krai [81], the Red Data Book of the Altai Republic [82], and the Red Data Book of China [83] were compiled where available. These conservation systems were compiled in parallel but were not treated as directly equivalent. IUCN categories are based on standardized global criteria concerning population decline, range size, population size, and extinction risk, whereas national and regional Red Data Books may reflect legal protection, regional rarity, restricted distribution, or expert assessment within a particular jurisdiction.

### 2.4. Comparative Floristic Analyses

Four incidence-based floristic datasets were used: the Koktau checklist compiled in the present study and published checklists for the Ulba Range [84], West Altai Nature Reserve [85], and Bukhtarma Mountains [86]. Before comparison, names from the four checklists were matched to POWO, synonymous records were consolidated, and accepted species- and infraspecific-level entries were used as the terminal units. Differences in the original taxonomic scope and inventory completeness may nevertheless remain; the resulting comparisons are therefore descriptive. These three regions were selected because they are geographically adjacent or regionally relevant phytogeographical units of the Kazakhstan Altai for which sufficiently comprehensive vascular-plant checklists were available. Together, they represent contrasting mountain-taiga, forest, meadow-steppe, and steppe-associated conditions. Each accepted taxon was coded as present (1) or absent (0) in each region. Jaccard similarity [87] was selected because the datasets consisted of presence–absence records without comparable abundance data and because the coefficient excludes joint absences. The corresponding dissimilarity matrix was calculated as 1 − J and used to construct an exploratory UPGMA dendrogram. The suggested binary Bray–Curtis coefficient is equivalent to the Sørensen–Dice coefficient for presence–absence data; Jaccard was retained to maintain consistency between the pairwise similarity table and the cluster analysis. The analyses and exploratory dendrogram were produced in R version 4.5.2 [88]. Because the regional inventories differ in area, taxonomic scope, and survey history, the coefficients and clustering were interpreted descriptively, and no inferential significance testing was applied. Spearman’s rank correlation coefficient was used descriptively to compare the rankings of the leading families among regional floras. No inferential significance testing was applied because the analysis was based on a limited set of ranked families.

### 2.5. Floristic Autonomy Index

The floristic autonomy index was included as a complementary descriptor of taxonomic structure because compositional similarity coefficients such as Jaccard quantify shared taxa but do not indicate whether richness is concentrated within relatively few genera or distributed among numerous genera. The index was retained solely as a supplementary descriptive measure of species–genus structure and was not used to infer the historical origin, degree of autochthony or allochthony, or assembly processes of the compared floras. The floristic autonomy index was calculated following Malyshev [89,90] for the complete vascular plant checklist of each region:

$$A=\;\frac{S-\;S_{x}}{S}$$

where S is the observed number of taxa and $S_{x}$ is the expected number estimated from the observed number of genera:

$$S_{x}=314.1+0.0045383\times G^{2}$$

where *G* is the observed number of genera. The constants are not fitted to the present data; they are the intercept and quadratic coefficient of the empirical species–genus relationship derived by Malyshev from a comparative dataset of regional floras. The index compares observed taxon richness with the richness expected from generic richness. Positive values indicate that the observed taxon richness exceeds the value expected from generic richness, whereas negative values indicate the reverse. The method does not define universal threshold classes or provide an associated measure of uncertainty. Consequently, the values were reported only as descriptive characteristics of species–genus structure, and no conclusions concerning floristic origin or assembly processes were drawn from their sign or magnitude. The index was calculated using all accepted taxa in each regional checklist and does not explicitly correct for differences in sampling effort or inventory completeness.

## 3. Results

### 3.1. Taxonomic, Ecological, and Growth-Form Structure of the Flora of the Koktau Mountains

**Figure 3:**
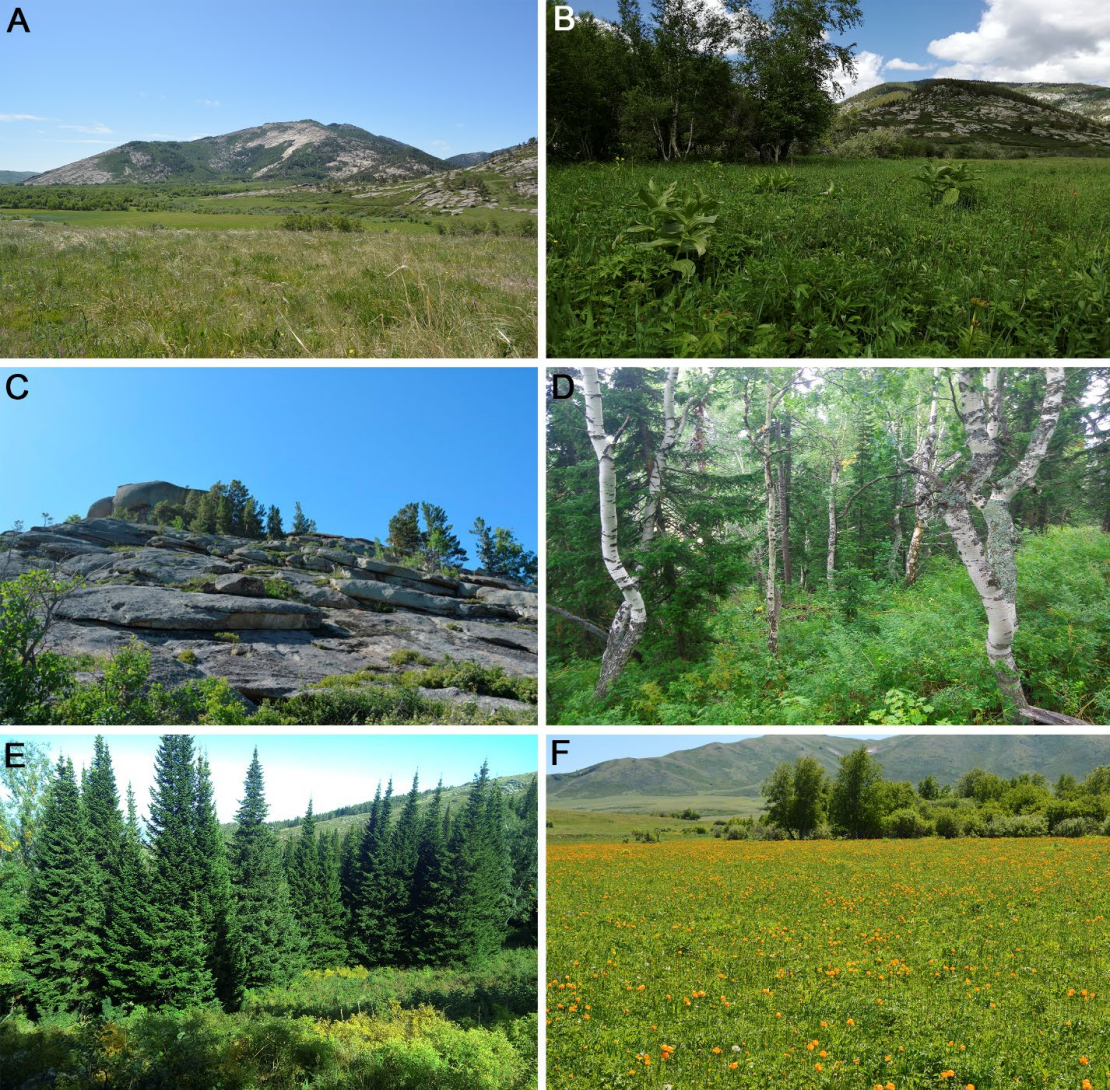
Primary relief features and vegetation communities of the Koktau Mountains: (**A**) feather-grass steppe, (**B**) marshy meadows, (**C**) woolsack-like (mattress-like) granitoids, (**D**) mixed forests, (**E**) fir stands, and (**F**) herb-rich meadows. All photographs were taken by the authors.

The relief of Koktau mountains is characterized by dissected granitic massifs, intermountain depressions, river valleys, and numerous rocky outcrops (Figure 3).

The central part of the mountain system includes the Sibin Depression with a network of small rivers and lakes. The vegetation cover consists of forest, meadow, and steppe communities, while forest vegetation is mainly confined to river valleys, ravines, and north-facing slopes.

In this study, “taxon” denotes an accepted terminal checklist entry at either species or infraspecific rank, including subspecies and varieties. The taxonomic structure of the flora is characteristic of temperate Holarctic regions and generally resembles that of neighboring phytogeographical regions within the Kazakhstan Altai (Table 1).

The vascular flora of the Koktau Mountains comprises 817 accepted taxa at species and infraspecific ranks, belonging to 356 genera and 79 families (Table S1). Five species—*Carex pediformis*, *Dactylorhiza majalis*, *Betula pendula*, *Hesperis sibirica*, and *Erigeron acris*—were represented by both species-level and additional infraspecific entries, including subspecies or varieties. Angiosperms constituted the dominant component of the flora, comprising 788 taxa distributed among 342 genera and 73 families and accounting for 96.5% of all recorded taxa. Within the angiosperms, eudicots outnumbered monocots by nearly threefold. Ferns comprised 22 accepted taxa belonging to 10 genera and three families, whereas gymnosperms comprised seven taxa belonging to four genera and three families. Lycophytes were not recorded in the Koktau Mountains.

The taxonomic structure of the Koktau flora closely resembles that of neighboring phytogeographical regions of the Kazakhstan Altai (Table 1). Across all compared regions, angiosperms represent the overwhelmingly dominant component of the flora, while the contribution of gymnosperms, ferns, and lycophytes remains relatively small. The Ulba Range exhibited the highest overall taxonomic richness, comprising 1015 taxa, 420 genera, and 100 families, whereas the West Altai Nature Reserve showed the greatest diversity of lycophytes and ferns.

**Figure 4:**
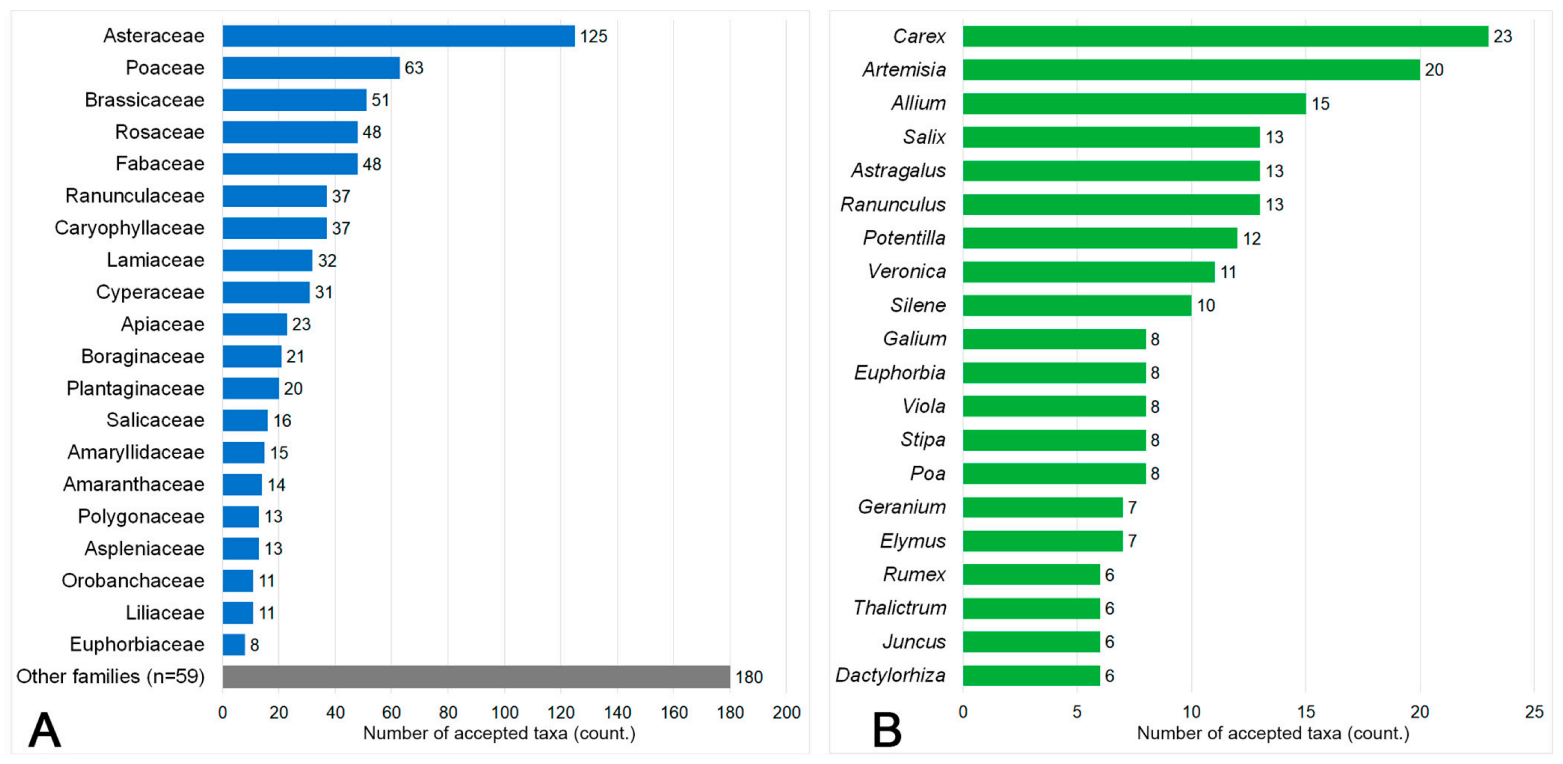
Taxonomic structure of the Koktau vascular flora. (**A**) Distribution of all 817 accepted taxa among the 20 most taxon-rich families, with the remaining 59 families combined as “Other families”; (**B**) the 20 most taxon-rich genera, collectively containing 208 accepted taxa. The horizontal axes show the number of accepted species- and infraspecific-level checklist entries.

The taxonomic structure of the Koktau flora is characterized by a pronounced concentration of species within a relatively small number of families and genera (Figure 4).

Fourteen families were represented by more than 15 taxa each and constituted the most taxon-rich component of the recorded flora. The most species-rich family was Asteraceae (125 species), accounting for more than 15% of the total flora, followed by Poaceae (63 species), Brassicaceae (51 species), Fabaceae (48 species), and Rosaceae (48 species). Other well-represented families included Caryophyllaceae and Ranunculaceae (37 species each), Lamiaceae (32 species), Cyperaceae (31 species), and Apiaceae (23 species) (Figure 4A). Together, these families comprise a substantial proportion of the total species diversity recorded in the Koktau Mountains.

At the generic level, species richness was distributed among numerous genera, although several were notably prominent. The largest genus was *Carex* with 23 species, followed by *Artemisia* (20 species) and *Allium* (15 species). Genera represented by more than ten species included *Astragalus*, *Ranunculus*, and *Salix* (13 species each), *Potentilla* (12 species), *Veronica* (11 species), and *Silene* (10 species) (Figure 4B). The predominance of these genera reflects the heterogeneous environmental conditions of the study area, encompassing meadow, steppe, wetland, and rocky habitats.

**Figure 5:**
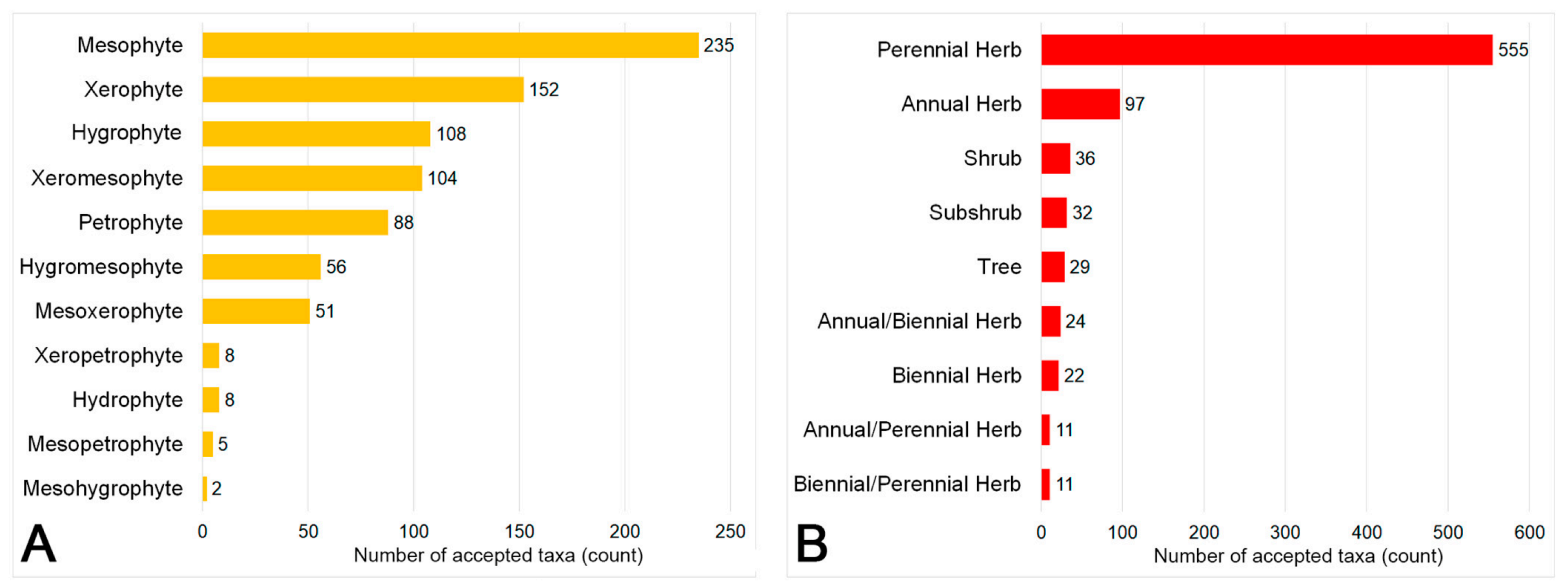
Ecological and growth-form composition of the Koktau vascular flora based on all 817 accepted taxa. (**A**) Distribution among ecological groups according to moisture and substrate preferences; (**B**) distribution among growth-form categories. Values shown on the bars indicate the absolute number of taxa assigned to each category.

Analysis of ecological groups revealed a broad spectrum of habitat preferences among the recorded species (Figure 5A).

Mesophytes formed the largest ecological group with 235 species, representing approximately 29% of the flora. Xerophytes were the second-most abundant group (152 species), followed by hygrophytes (108 species), xeromesophytes (104 species), and petrophytes (88 species). Hygromesophytes (56 species) and mesoxerophytes (51 species) were moderately represented, whereas hydrophytes, xeropetrophytes, mesopetrophytes, and mesohygrophytes collectively accounted for only a small fraction of the flora. The simultaneous prominence of mesophytic and xerophytic species reflects the marked environmental heterogeneity of the Koktau Mountains, where moist river valleys and lakeshores occur in close proximity to dry rocky slopes and steppe habitats.

The growth-form spectrum was strongly dominated by herbaceous plants (Figure 5B). Perennial herbs constituted the largest group with 555 species, representing more than two-thirds of all recorded taxa. Annual herbs formed the second-largest category with 97 species, while shrubs and subshrubs were represented by 36 and 32 species, respectively. Trees accounted for only 29 species. Biennial herbs (22 species) and transitional growth-form categories, including annual/biennial, annual/perennial, and biennial/perennial herbs, were comparatively rare. Overall, the predominance of perennial herbaceous species indicates the decisive role of meadow-steppe and mountain grassland vegetation in shaping the floristic composition of the Koktau Mountains.

### 3.2. Rare and Threatened Species

**Figure 6:**
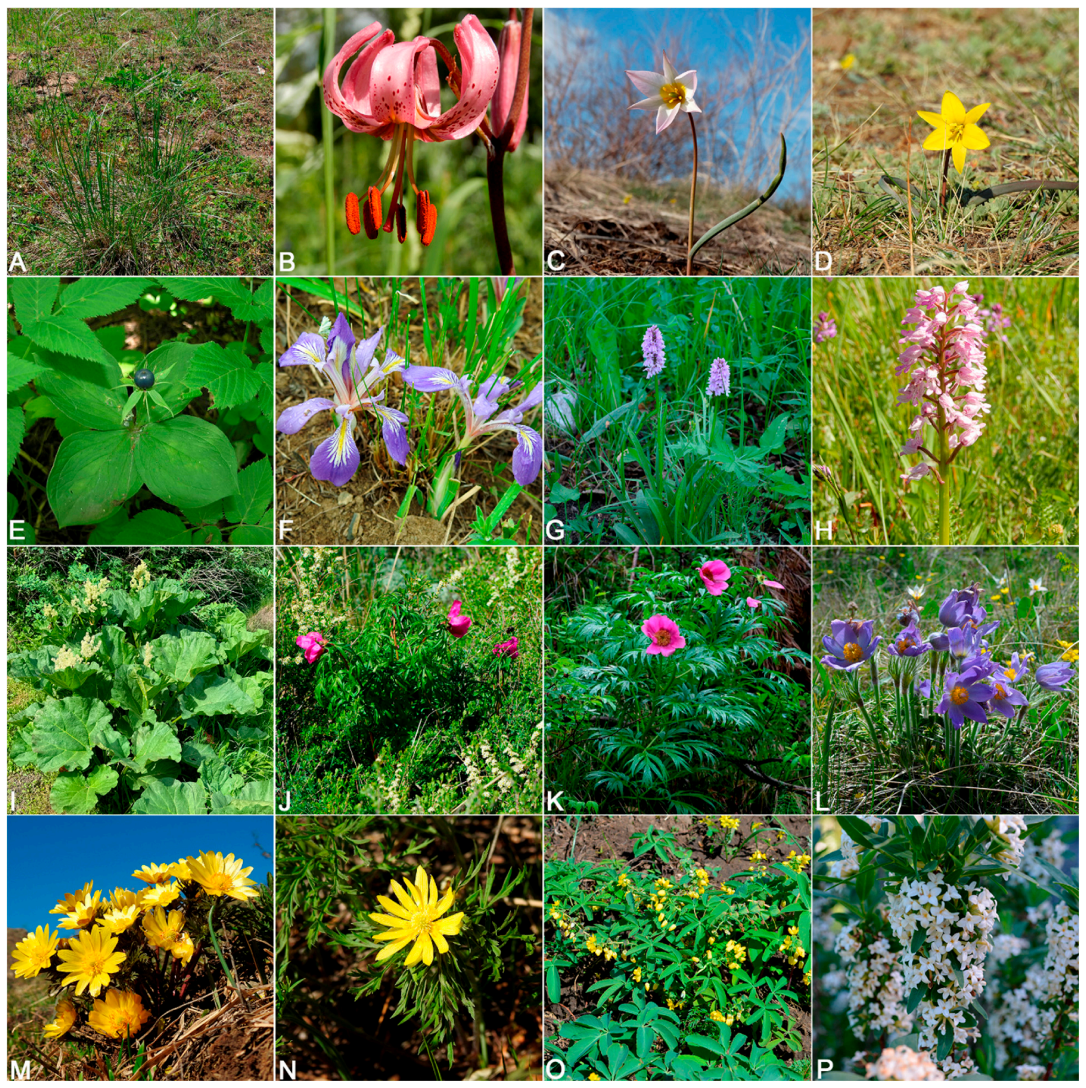
Red Data Book plant species recorded in the Koktau Mountains: (**A**) *Stipa pennata*; (**B**) *Lilium martagon*; (**C**) *Tulipa sylvestris* subsp. *australis*; (**D**) *Tulipa uniflora*; (**E**) *Paris quadrifolia*; (**F**) *Iris ludwigii*; (**G**) *Dactylorhiza fuchsii*; (**H**) *Orchis militaris*; (**I**) *Rheum compactum*; (**J**) *Paeonia tenuifolia*; (**K**) *Paeonia anomala*; (**L**) *Pulsatilla patens*; (**M**) *Adonis villosa*; (**N**) *Adonis volgensis*; (**O**) *Gymnospermium altaicum*; and (**P**) *Daphne altaica*. All photographs were taken by the authors.

Comprehensive surveys conducted throughout the entire vegetation period resulted in the identification of 16 species included in the Red Data Book of Kazakhstan (Figure 6, Table 2). Several of these species are early-flowering geophytes and spring ephemeroids, including *Tulipa sylvestris* subsp. *australis*, *Tulipa uniflora*, *Pulsatilla patens*, *Adonis villosa*, *Adonis volgensis*, and *Gymnospermium altaicum*. The recorded protected species represent a broad range of taxonomic groups and ecological habitats, including steppe, meadow, forest, and rocky-slope communities.

Among the recorded species, the majority belong to categories “Very rare species” and “Rare species with declining populations” of the Red Data Book of Kazakhstan, corresponding to very rare species and rare species with declining populations, respectively (Table 2). Category “Very rare species” included *Lilium martagon*, *Paris quadrifolia*, *Dactylorhiza fuchsii*, *Pulsatilla patens*, *Adonis villosa*, and *Daphne altaica*, whereas category “Rare species with declining populations” comprised eight species. Two taxa, *Paeonia anomala* and *Gymnospermium altaicum*, were assigned to category “Species of uncertain status”.

Compilation of international, national, and regional conservation assessments showed that several Koktau taxa are recognized as conservation-relevant at more than one geographical scale. Because these systems differ in scope and assessment criteria, their categories are reported in parallel but are not interpreted as directly equivalent. The IUCN Data Deficient category indicates insufficient information for assessing extinction risk and is not itself a threatened category. *Tulipa uniflora*, *Orchis militaris*, *Rheum compactum*, *Paeonia tenuifolia*, and *Daphne altaica* are included in regional Red Lists of neighboring countries and territories, including Russia and China, and have also been assessed by the IUCN Red List under categories including Near Threatened (NT) to Vulnerable (VU) and Data Deficient (DD) (Table 2). In particular, *Tulipa uniflora* and *Daphne altaica* are classified as Vulnerable in China, while *Orchis militaris* and *Rheum compactum* are listed as Endangered in China. Several species, including *Paeonia tenuifolia* and *Lilium martagon*, are also regarded as endangered within the Kazakhstan Altai.

### 3.3. Regional Floristic Richness and Phytogeographical Relationships

The checklist of the Koktau Mountains contains 817 accepted taxa recorded within an area of approximately 510 km^2^ (Table 3).

Despite covering a substantially smaller area, the Koktau flora approaches the total species richness of the Bukhtarma Mountains (854 taxa), which occupy an area more than ten times larger. A total of 108 medicinal plant species were documented in the study area. Twenty-seven rare and threatened taxa were recorded, a slightly lower number than reported for the neighboring regions, where the number of protected species ranges from 30 to 33. Two endemic species of Kazakhstan, *Astragalus chaetolobus* Bunge and *Elymus sibinicus* Kotukhov, were identified within the study area. Notably, *E. sibinicus* represents a narrow local endemic currently known only from the Koktau Mountains. No endemic genera were recorded.

To assess floristic relationships among regional floras, Jaccard similarity coefficients were calculated based on species composition (Table 4).

**Figure 7:**
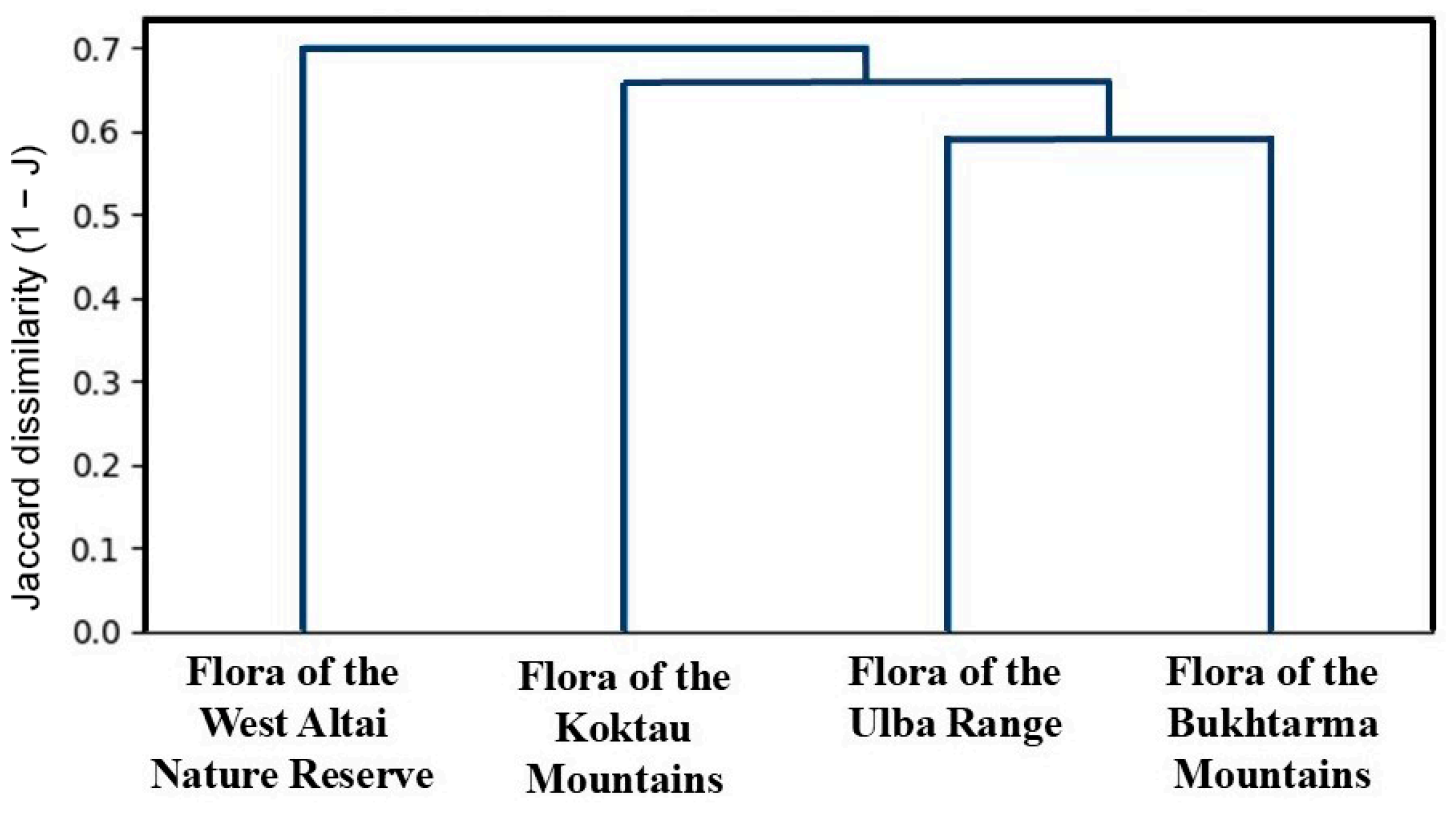
Exploratory UPGMA dendrogram constructed in R version 4.5.2 from Jaccard dissimilarities (1 − J) among the complete vascular-plant checklists of the four compared regions. The vertical axis shows Jaccard dissimilarity, and the terminal labels identify the regional floras. The clustering is descriptive; branch support was not inferred because the inventories differ in area and survey history.

The Koktau flora exhibited the highest similarity to the Bukhtarma Mountains (0.354) and the Ulba Range (0.328), whereas the lowest similarity was observed with the West Altai Nature Reserve (0.249). The latter region is characterized predominantly by mountain-taiga vegetation, in contrast to the forest-steppe and meadow-steppe communities prevailing in the Koktau Mountains. Among all compared regions, the strongest floristic affinity was found between the Ulba Range and the Bukhtarma Mountains (0.409). Cluster analysis further illustrated these relationships (Figure 7).

The dendrogram grouped the floras of the Ulba Range and Bukhtarma Mountains into a closely related cluster, while the Koktau flora was associated with this group at a slightly greater distance. In contrast, the flora of the West Altai Nature Reserve formed the most distinct branch, reflecting its unique taxonomic composition and ecological differentiation from the other regional floras.

The checklist included numerous ruderal and weed species, including *Chelidonium majus*, *Sophora alopecuroides*, *Cannabis sativa*, *Humulus lupulus*, *Urtica cannabina*, *U. dioica*, *U. urens*, *Ecballium elaterium*, *Acer negundo*, *Polygonum aviculare*, *Atriplex tatarica*, *Chenopodium album*, *Calystegia sepium*, *Cuscuta europaea*, *C. lupuliformis*, *Solanum dulcamara*, *Xanthium strumarium*, and *Conium maculatum* (Table S1). The ten largest families collectively contained 495 species, accounting for 60.6% of the flora, compared with 58.4% in the Bukhtarma Mountains, 57.7% in the West Altai Nature Reserve, and 57.2% in the Ulba Range (Table 5).

The taxonomic structure of the leading families was generally similar among all compared floras. However, several distinctive features characterize the Koktau flora. The family Asteraceae occupies a particularly dominant position, comprising 125 species (15.3% of the total flora), nearly twice as many as Poaceae, which ranks second with 63 species (7.7%). This pattern closely resembles that observed in the Ulba Range, whereas Poaceae is the most species-rich family in both the Bukhtarma Mountains and the West Altai Nature Reserve. Another notable feature is the relatively high representation of Caryophyllaceae, which accounts for 4.53% of the Koktau flora. Its contribution is comparable to that of the Ulba Range but exceeds that recorded for the Bukhtarma Mountains (3.51%) and the West Altai Nature Reserve (3.40%).

The similarity of family spectra among regional floras was evaluated using Spearman’s rank correlation coefficient (Table 6).

Correlation values ranged from 0.818 to 0.915 between the Koktau flora and neighboring regions, indicating a high degree of consistency in the ranking of dominant families. Likewise, the Koktau flora showed strong agreement with the overall floristic spectrum of the Kazakhstan Altai (ρ = 0.927), indicating descriptive similarity between its leading-family spectrum and that of the broader Kazakhstan Altai flora.

The calculated floristic autonomy index values for the four regional checklists ranged from −0.147 in the Bukhtarma Mountains to −0.042 in the West Altai Nature Reserve (Table 7).

The value calculated for the Koktau Mountains was −0.088, compared with −0.098 for the Ulba Range, −0.042 for the West Altai Nature Reserve, and −0.147 for the Bukhtarma Mountains. These values are presented solely as descriptive characteristics of species–genus structure, and no inference concerning floristic origin or assembly processes is made from their sign or magnitude.

Overall, the comparative analyses demonstrate that the Koktau flora combines substantial taxonomic richness with clear floristic affinities to neighboring regions. These results emphasize the important role of the Koktau Mountains in maintaining regional plant diversity and preserving unique floristic elements within the Kazakhstan Altai.

## 4. Discussion

The comprehensive floristic inventory presented in this study reveals the Koktau Mountains as a floristically rich area within the Kalba Ridge and confirms their important contribution to the overall floristic diversity of the Kazakhstan Altai. By integrating extensive original field collections (more than 2700 herbarium specimens gathered between 2018 and 2025) with a critical synthesis of materials from the herbaria ABG, AA, LE, and MW, this work addresses a long-standing knowledge gap and provides the first detailed checklist of the vascular flora of the Koktau Mountains. In addition, it clarifies the taxonomic structure, ecological differentiation, and phytogeographic position of the flora within the broader Altai-Sayan system. The vascular flora of the Koktau Mountains comprises 817 taxa belonging to 356 genera and 79 families within a relatively small area of approximately 510 km^2^ (Table S1). Taxon density is inherently scale-dependent and is influenced by study-area size, habitat heterogeneity, accessibility, survey duration, and inventory intensity. Because the surveys were designed as a floristic inventory rather than as standardized habitat-level sampling, the relative contributions of these factors cannot be partitioned quantitatively. The value reported for the Koktau Mountains should therefore be treated descriptively, reflecting both intensive multi-year sampling and genuine habitat heterogeneity, rather than as evidence that the region is intrinsically more diverse than larger territories. In addition, the transitional position of the Koktau Mountains promotes the overlap of boreal, mountain, steppe, Mediterranean, and Turanian floristic elements, producing a species-rich assemblage without a correspondingly high level of endemism. The comparatively high density of the smaller West Altai Nature Reserve further indicates that area alone does not explain the observed regional differences. In particular, the mosaic of granitic outcrops, intermountain basins, and river valleys creates a wide range of microhabitats that likely promote species coexistence. These patterns underscore the Koktau Mountains’ role as a local center of floristic richness [2] and a transitional territory linking the mountain systems of the Altai with the steppe landscapes of Eastern Kazakhstan.

The taxonomic structure is typical of temperate Holarctic mountain floras. Lycophytes were not recorded in the Koktau Mountains, whereas three species are known from the West Altai Nature Reserve [42]. This difference may reflect the lower elevation of the Koktau Mountains and the limited availability of cool, persistently moist mountain-taiga and high-mountain habitats typically occupied by lycophytes in the Kazakhstan Altai. Nevertheless, because some potentially suitable microsites may have been difficult to access, incomplete detection cannot be entirely excluded. The leading families—Asteraceae (125 species), Poaceae (63), Brassicaceae (51), Fabaceae (48), and Rosaceae (48) (Figure 4A)—closely align with patterns reported from other regions of the Kazakhstan Altai and Central Asia [7,42]. However, the exceptionally high contribution of Asteraceae in the Koktau Mountains (15.3% of the flora) exceeds that recorded in the Bukhtarma Mountains (9.36%), West Altai Nature Reserve (12.60%), and Ulba Range (12.71%), reflecting the transitional position of the Kalba. Potential sampling effects should nevertheless be considered when interpreting the taxonomic spectrum. Differences in phenology, detectability, habitat accessibility, and taxonomic complexity may influence the recorded richness of individual families and genera. Repeated surveys across several vegetation seasons and verification of voucher specimens against four herbarium collections reduced these effects but could not eliminate them completely. The high richness of *Carex* is ecologically consistent with the diversity of wet meadows, lakeshores, river valleys, and marshy habitats within the Sibin Depression [91]. Similarly, the predominance of Asteraceae is broadly consistent with other steppe-influenced floras of the Kazakhstan Altai, although some influence of collection and identification effort cannot be excluded [92].

At the generic level, the spectrum shows a characteristic mixture of boreal northern genera (*Carex*—23 species, *Salix*, *Ranunculus*) and southern montane/Central Asian genera (*Artemisia*—20, *Allium*—15, *Astragalus*, *Stipa*) (Figure 4B). This duality reflects the geographic position of the Koktau Mountains at the intersection of floristic influences from the Narym Ridge (northeast), Kazakh Uplands (southwest), and Zaysan steppes (southeast). The resulting overlap of floristic elements enhances species richness but reduces regional uniqueness, supporting the interpretation of the Koktau Mountains as a transitional floristic territory.

Ecologically, the flora demonstrates considerable heterogeneity, with mesophytes (235 species) and xerophytes (152 species) prevailing, followed by hygrophytes (108) and petrophytes (88) (Figure 5A). This distribution reflects the strong environmental gradients in moisture availability, soil composition, and elevation. The relatively high proportion of hygrophytic and petrophytic species is particularly indicative of the importance of river valleys, wet meadow systems, and rocky granitic substrates. The dominance of perennial herbaceous plants (555 species) further emphasizes the importance of meadow-steppe and grassland communities in structuring the vegetation mosaic [93], highlighting the structural importance of meadow-steppe and grassland habitats in the recorded flora.

Floristic similarity analysis using the Jaccard coefficient revealed the highest similarity of the Koktau flora with the Bukhtarma Mountains (0.354) and Ulba Range (0.328), whereas the lowest value was observed for the West Altai Nature Reserve (0.249) (Table 4). These coefficients should be interpreted as descriptive because Jaccard similarity is sensitive to differences in species richness, study-area size, sampling intensity, and inventory completeness and may underestimate similarity when the compared floras differ substantially in richness. Effort-standardized rarefaction or abundance-based similarity measures could not be applied because comparable occurrence-level and sampling-effort data were unavailable for the published regional floras. The UPGMA dendrogram should likewise be regarded as an exploratory representation of the similarity matrix. The closer grouping of the Ulba Range and Bukhtarma Mountains may reflect their greater floristic continuity and shared mountain-forest, meadow, and steppe-associated elements. The Koktau flora shares many of these elements but is more strongly influenced by lower-elevation forest-steppe and dry-steppe habitats. In contrast, the distinct position of the West Altai Nature Reserve is consistent with its stronger mountain-taiga character and its comparatively greater representation of ferns and lycophytes. These ecological interpretations remain provisional because the regional inventories differ in area and sampling history. These explanations should be regarded as hypotheses generated by the checklist comparison and require evaluation using standardized sampling across the four regions.

The documentation of 27 rare and threatened taxa, including 16 species listed in the Red Data Book of Kazakhstan, highlights the high conservation significance of the Koktau Mountains (Table 2). Species such as *Tulipa uniflora*, *Daphne altaica*, *Orchis militaris*, and *Rheum compactum* represent important conservation targets because of their regional rarity and ecological specialization. Particular attention should be given to *Elymus sibinicus* Kotukhov, a narrow local endemic currently known only from the Koktau Mountains. According to the habitat information compiled in Table S1, this perennial mesophytic grass occurs in coastal meadows with mixed grasses and shrubs. However, the present floristic inventory was not designed to estimate its number of populations, population size, demographic structure, or temporal trends. These parameters and the effects of potential habitat disturbance therefore require targeted field assessment. The occurrence of only two species endemic to Kazakhstan—*Astragalus chaetolobus* and *E. sibinicus*—is consistent with the transitional and weakly isolated position of the Koktau Mountains. Thus, the region is better interpreted as an area of floristic overlap and exchange than as a major center of in situ speciation, although localized habitats may still support narrow endemic taxa.

The results support several practical conservation priorities. First, all known localities of *Elymus sibinicus* and nationally protected species should be georeferenced and incorporated into a long-term population-monitoring program. Second, conservation measures should focus not only on individual species but also on the principal habitats supporting floristic diversity, particularly the wetland and riparian systems of the Sibin Depression, moist forested ravines, meadow-steppe communities, and granitic outcrops. Third, permanent monitoring sites should be established to detect changes in rare-species populations, habitat condition, and the spread of ruderal taxa. The checklist and distribution records generated in this study can also support environmental impact assessment, regional land-use planning, and a future evaluation of the Koktau Mountains under the Global KBA Standard [94] and regional protected-area criteria. The present study does not establish Key Biodiversity Area status; formal designation would require criterion-specific population, distribution, threat, and land-use evidence.

However, the Red Data Book of Kazakhstan provides the national legal conservation status, whereas the Kazakhstan Altai checklist evaluates rarity within the Kazakhstan portion of the Altai. The Red Lists of the IUCN, China, Altai Krai, and the Altai Republic provide global and neighboring regional perspectives. These assessments are therefore complementary and may assign different categories to the same species because of differences in geographical scope and assessment criteria.

The relative effects of grazing, recreation, roads, settlements, agriculture, fire, mining, or other potential pressures cannot be quantified from the available data and should not be inferred solely from the presence of ruderal taxa. Likewise, the concentration of 60.6% of the flora within the ten leading families differs only moderately from the corresponding values of the comparison regions and should not be regarded as independent evidence of vegetation homogenization. Establishing whether homogenization is occurring will require repeated vegetation surveys combined with spatially explicit information on land use, disturbance, and the temporal dynamics of specialist and generalist species [95]. Their occurrence indicates potential anthropogenic influence but does not quantify the extent, intensity, or temporal development of disturbance.

## 5. Conclusions

The Koktau flora is characterized by the coexistence of boreal, steppe, Mediterranean, and Turanian elements across forest, meadow, wetland, steppe, and rocky habitats. Its greatest compositional affinity with the Bukhtarma Mountains and Ulba Range, together with low endemism and the overlap of multiple floristic elements, is consistent with the hypothesis that the Koktau Mountains function as a transitional area of floristic exchange, whereas testing the relative contribution of in situ diversification would require phylogenetic and population-level evidence. Nevertheless, the occurrence of 16 nationally protected species and the local endemic *Elymus sibinicus* establishes clear conservation priorities. The checklist provides a baseline for conservation planning, environmental assessment, and long-term monitoring. The principal limitations are the absence of standardized plot-based sampling, population-demographic data, and temporal habitat assessments. Future work should prioritize demographic monitoring of *E. sibinicus* and nationally protected species, standardized vegetation surveys, and investigation of bryophytes, lichens, and fungi.

## Figures and Tables

**Table 1:** Taxonomic composition of the vascular flora of the Koktau Mountains and neighboring phytogeographical regions of the Kazakhstan Altai.

Regions	Major Taxonomic Groups	Number of Families	Number of Genera	Number of Taxa
Koktau mountains	Lycophytes	0	0	0
	Ferns	3	10	22
	Gymnosperms	3	4	7
	Angiosperms	73	342	788
	Total	79	356	817
Ulba Range	Lycophytes	0	0	0
	Ferns	11	12	20
	Gymnosperms	3	6	11
	Angiosperms	86	402	984
	Total	100	420	1015
West Altai Nature Reserve	Lycophytes	2	3	3
	Ferns	9	13	32
	Gymnosperms	3	6	13
	Angiosperms	70	316	731
	Total	84	338	799
Bukhtarma Mountains	Lycophytes	0	0	0
	Ferns	9	12	29
	Gymnosperms	3	6	9
	Angiosperms	84	365	816
	Total	96	383	854

**Table 2:** Rare and threatened vascular plant species recorded in the Koktau Mountains and their conservation status according to the Red Data Book of Kazakhstan, the IUCN Red List, the Kazakhstan Altai checklist of rare species, and regional Red Lists of Russia and China.

Taxon	Red Data Book of Kazakhstan	IUCN Global Category	Kazakhstan Altai Regional Status	Red Data Book of Altai Krai (Russia) ^1^	Red Data Book of the Altai Republic (Russia) ^2^	China Threatened-Species Status
*Stipa pennata*	Rare species with declining populations	–	–	3c	–	–
*Lilium martagon*	Very rare species	Least concern	Endangered	–	–	–
*Tulipa sylvestris* subsp. *australis*	Rare species with declining populations		Near threatened	3b		
*Tulipa uniflora*	Rare species with declining populations	Near threatened	Vulnerable	2b		Vulnerable
*Paris quadrifolia*	Very rare species	Least concern	–	–	–	–
*Iris ludwigii*	Rare species with declining populations	–	Vulnerable	2a		
*Dactylorhiza fuchsii*	Very rare species	Least concern	Vulnerable		3	
*Orchis militaris*	Rare species with declining populations	Least concern	Near threatened	3b	3	Endangered
*Rheum compactum*	Rare species with declining populations	–	Vulnerable	2b	3	Endangered
*Paeonia tenuifolia*	Rare species with declining populations	Data deficient	Endangered	3a	3	–
*Paeonia anomala*	Species of uncertain status	–	Vulnerable	–	–	Vulnerable
*Pulsatilla patens*	Very rare species	Data deficient	–	–	–	–
*Adonis villosa*	Very rare species	–	–	–	3	–
*Adonis volgensis*	Rare species with declining populations	–	–	2c	–	–
*Gymnospermium altaicum*	Species of uncertain status	–	–	3a	3	–
*Daphne altaica*	Very rare species	Data deficient	Vulnerable	–	2	Vulnerable

^1^ 2a—vulnerable narrow-range endemics of Altai Krai whose distribution is confined to the Greater Altai or extends only slightly beyond its borders; 2b—vulnerable species with restricted distributions occurring in Russia exclusively within the Altai region; 2c—vulnerable species with broader geographic ranges that are extremely rare and particularly vulnerable within Altai Krai; 3a—rare endemic species of the Altai Mountain Country; 3b—rare species with wide geographic ranges occurring in specific habitats; 3c—rare species included in the Red Data Book of the Russian Federation and protected under the regional conservation system. ^2^ 2—vulnerable species likely to become endangered if limiting factors continue to operate; 3—rare species represented by small populations that are currently neither endangered nor vulnerable but are at risk of becoming so in the future.

**Table 3:** Floristic diversity of the Koktau Mountains and neighboring phytogeographical regions of the Kazakhstan Altai.

Phytogeographic Region	Area, km^2^	Number of Taxa		Number of Medicinal Species	Number of Rare and Endangered Species	Number of Endemics
		Total	Per 1 km^2^ (Species Density)			
Koktau Mountains	510	817	1.60	108	27	2
Ulba Range	3220	1015	0.31	340	33	2
West Altai Nature Reserve	861	799	0.93	299	30	0
Bukhtarma Mountains	5308	854	0.16	285	32	2

Note: Taxon density is presented as a descriptive ratio of checklist richness to area. It is scale-dependent and is not standardized for habitat heterogeneity, accessibility, survey duration, or inventory intensity. Medicinal-plant counts are reproduced from the corresponding regional floristic sources and are presented descriptively.

**Table 4:** Pairwise Jaccard similarity coefficients among the vascular floras of the Koktau Mountains and the three compared regions.

Phytogeographic Region	Koktau Mountains	Ulba Range	West Altai Nature Reserve	Bukhtarma Mountains
Koktau Mountains (n = 817)	1	0.328	0.249	0.354
Ulba Range (n = 1015)	0.328	1	0.323	0.409
West Altai Nature Reserve (n = 799)	0.249	0.323	1	0.326
Bukhtarma Mountains (n = 854)	0.354	0.409	0.326	1

**Table 5:** Composition of the ten leading plant families in the flora of the Koktau Mountains and neighboring phytogeographical regions.

Family	Flora of the Koktau Mountains		Flora of the Bukhtarma Mountains		Flora of the West Altai Nature Reserve		Flora of the Ulba Range	
	Number of Species/Percentage of Total Species	Number of Genera/Percentage of Total Genera	Number of Species/Percentage of Total Species	Number of Genera/Percentage of Total Genera	Number of Species/Percentage of Total Species	Number of Genera/Percentage of Total Genera	Number of Species/Percentage of Total Species	Number of Genera/Percentage of Total Genera
Asteraceae	125/15.30%	54/15.17%	80/9.36%	40/10.44%	100/12.60%	45/13.31%	129/12.71%	52/12.38%
Poaceae	63/7.71%	33/9.27%	88/10.30%	37/9.66%	101/12.70%	32/9.47%	75/7.39%	36/8.57%
Brassicaceae	51/6.24%	33/9.27%	44/5.15%	28/7.31%	34/4.30%	20/5.92%	54/5.32%	28/6.66%
Fabaceae	48/5.88%	14/3.93%	76/8.89%	17/4.43%	37/4.50%	11/3.25%	68/6.70%	14/3.33%
Rosaceae	48/5.88%	20/5.62%	47/5.50%	18/4.69%	45/5.70%	18/5.32%	51/5.02%	21/5.00%
Caryophyllaceae	37/4.53%	13/3.61%	30/3.51%	14/3.65%	27/3.40%	14/4.14%	46/4.53%	21/5.00%
Ranunculaceae	37/4.53%	10/2.81%	42/4.91%	16/4.17%	34/4.30%	15/4.43%	52/5.12%	20/4.76%
Lamiaceae	32/3.92%	16/4.49%	22/2.57%	15/3.91%	25/3.10%	14/4.14%	33/3.25%	19/4.52%
Cyperaceae	31/3.79%	7/1.97%	36/4.21%	9/2.34%	34/4.30%	5/1.48%	39/3.84%	5/1.19%
Apiaceae	23/2.82%	17/4.77%	34/3.98%	19/4.96%	22/2.80%	16/4.73%	34/3.35%	24/5.71%
Total	495/60.60%	217/60.95%	499/58.38%	213/55.61%	459/57.70%	190/56.2%	581/57.23%	240/57.14%

**Table 6:** Spearman rank correlation coefficients (ρ) for the spectra of leading plant families among the compared phytogeographical regions and the Kazakhstan Altai Mountains (KAM).

Phytogeographic Region	Koktau Mountains	Ulba Range	West Altai Nature Reserve	Bukhtarma Mountains	Kazakhstan Altai Mountains
Koktau Mountains	-	0.915	0.879	0.818	0.927
Ulba Range	0.915	-	0.855	0.915	0.964
West Altai Nature Reserve	0.879	0.855	-	0.939	0.927
Bukhtarma Mountains	0.818	0.915	0.939	-	0.939
KAM	0.927	0.964	0.927	0.939	-

**Table 7:** Floristic autonomy index and taxonomic characteristics of the compared phytogeographical regions.

Regions	Number of Taxa	Number of Genera	Number of Families	Mean Number of Species Per Genus	Autonomy Index
Koktau Mountains	817	356	79	2.29	−0.088
Ulba Range	1015	420	100	2.41	−0.098
West Altai Nature Reserve	799	338	84	2.36	−0.042
Bukhtarma Mountains	854	383	96	2.23	−0.147

Note: The autonomy index is presented solely as a descriptive measure of species–genus structure and is not interpreted as evidence of floristic origin or assembly history.

## Data Availability

All data generated or analyzed during this study are included in this published article and its Supplementary Materials.

## Supplementary Materials

The following supporting information can be downloaded at https://www.mdpi.com/article/10.3390/biology15151309/s1, Table S1: The complete checklist of the vascular flora of the Koktau Mountains.

## Abbreviations

The following abbreviations are used in this manuscript:

ABG	Altai Botanical Garden
APG	Angiosperm Phylogeny Group
IUCN	International Union for Conservation of Nature
KAM	Kazakhstan Altai Mountains
POWO	Plants of the World Online
UPGMA	Unweighted Pair Group Method with Arithmetic Mean
