# Supplementary material for: Vascular Plant Flora of the Koktau Mountains (Kalba Ridge): Diversity, Phytogeographical Relationships, and Conservation Significance in the Kazakhstan Altai
Source: Biology (Basel). 2026 Aug 5;15(15):1309. doi: 10.3390/biology15151309 (PMC13465060; doi:10.3390/biology15151309)
Supplement: Supplementary file 1 [file biology-15-01309-s001.zip › biology-4430812-supplementary.pdf]

Table S1 – The complete checklist of the vascular flora of the Koktau Mountains.

Taxon name	Life form	Ecomorph group	Ecology of the taxon's growth in the Koktau Mountains (Kazakhstan's Altai)
**Equisetaceae**			
Equisetum arvense L.	Perennial herbaceous	Hygrophyte	Along the banks of rivers, streams, in damp meadows, arable lands and fallow lands
E. fluviatile L.	Perennial herbaceous	Hygrophyte	Along the clay banks of rivers, lakes, damp and marshy meadows
E. hyemale L.	Perennial herbaceous	Hygrophyte	In floodplain forests, bushes, along river banks
E. pratense Ehrh.	Perennial herbaceous	Hygrophyte	Through valley meadows, in shrub thickets
E. ramosissimum Desf.	Perennial herbaceous	Xerophyte	On dry sandy places, river banks
E. sylvaticum L.	Perennial herbaceous	Hygrophyte	In valley forests, bush thickets, hummocky swamps
**Aspleniaceae**			
Asplenium ruprechtii Sa. Kurata	Perennial herbaceous	Mesophyte	On rocky outcrops in shrub forest communities, along wide mossy cracks: one location is known in the area of Lake Karakol.
A. ruta-muraria L.	Perennial herbaceous	Petrophyte	Along the cracks of well-lit mattress-like granitoids, mountain pine forests
A. septentrionale (L.) Hoffm.	Perennial herbaceous	Petrophyte	On well-lit, less often, with some shading, shale and mattress-like granitoids, less often, on other rocks: upland pine forests
A. trichomanes L.	Perennial herbaceous	Petrophyte	Along cracks in moderately shaded mattress-like granitoids:
Athyrium filix-femina (L.) Roth	Perennial herbaceous	Mesophyte	In the zone of moderately moist rocky pine forests, cliffs, north-east, north-west exposures, along the shaded bottoms of ravines.
Cystopteris dickieana R. Sim	Perennial herbaceous	Petrophyte	Along the cracks in the rocks, covered with moss, in the mountain pine forests
C. fragilis (L.) Bernh.	Perennial herbaceous	Petrophyte	On rocky substrates, in rocky pine forests
Gymnocarpium continentale (Petrov.) Pojark.	Perennial herbaceous	Petrophyte	Along the shaded deep cracks of mattress-like granitoids, under the cornices in mountain rocky pine forests
G. dryopteris (L.) Newman	Perennial herbaceous	Petrophyte	In mountain rocky communities of Pinus sylvestris
Onoclea struthiopteris (L.) Roth	Perennial herbaceous	Hygrophyte	Along damp forests and the edges of swamps:
Woodsia asiatica Kiselev & Shmakov	Perennial herbaceous	Petrophyte	Along cracks and cornices in mountain pine forests

W. calcarea (Fomin) Shmakov	Perennial herbaceous	Petrophyte	Along cracks and cornices of mattress-like granitoids
W. ilvensis (L.) R.Br.	Perennial herbaceous	Petrophyte	Along the outcrops of mattress-like granitoids that are part of the upland pine forests
**Polypodiaceae**			
Dryopteris filix-mas (L.) Schott	Perennial herbaceous	Petrophyte	In aspen groves, in rock depressions, in deep crevices in mountain pine forests
Polystichum braunii (Spenn.) Fee	Perennial herbaceous	Petrophyte	In the chern forests: one habitat is known (deep depression between rocks).
Polypodium vulgare L.	Perennial herbaceous	Petrophyte	Through the cracks in the rocks
**Pinaceae**			
Abies sibirica Ledeb.	Conifer	Xerophyte	On the north-western slopes, in deep shady gullies
Pinus sylvestris L.	Conifer	Xerophyte	On the rocky mountain ridges
**Cupressaceae**			
Juniperus communis var. *saxatilis* Pall.	Bush	Xerophyte	In the zone of plantings *of Abies sibirica* Ledeb
J . sabina L.	Bush	Xerophyte	On rocks and dry mountain slopes
**Ephedraceae**			
Ephedra equisetina Bunge	Bush	Xerophyte	On steppe rocky slopes, cliffs, in the steppe belt
E. intermedia Schrenk & CA Mey.	Bush	Xerophyte	Along the gravelly slopes, trails of steppe mountains and hills
E. monosperma JG Gmel. ex CA May.	Bush	Xerophyte	On dry slopes, in rocky pine forests
**Nymphaeaceae**			
Nymphaea candida C. Presl .	Perennial herbaceous	Hydrophyte	In lakes, rivers
**Acoraceae**			
Acorus calamus L.	Perennial herbaceous	Hygrophyte	Along the banks of reservoirs, swamps
**Alismataceae**			
Alisma plantago-aquatica L.	Perennial herbaceous	Hygrophyte	Along the banks of rivers, lakes, and damp meadows
Sagittaria natans Pall.	Perennial herbaceous	Hygrophyte	Along the banks of rivers and lakes
**Butomaceae**			
Butomus umbellatus L.	Perennial herbaceous	Hygrophyte	Along the river banks, damp meadows

**Hydrocharitaceae**			
Najas minor All.	Annual herbaceous	Hydrophyte	In shallow bays and oxbow lakes with fresh water
**Juncaginaceae**			
Triglochin palustris L .	Perennial herbaceous	Hygrophyte	Along damp river banks, damp bushy meadows, and the outskirts of swamps
**Potamogetonaceae**			
Potamogeton crispus L.	Perennial herbaceous	Hydrophyte	In stagnant or slow-flowing waters of lakes and river backwaters
P. lucens L.	Perennial herbaceous	Hydrophyte	In rivers, lakes
P. obtusifolius Mert. & W.D.J. Koch	Perennial herbaceous	Hydrophyte	In the lakes
P. perfoliatus L.	Perennial herbaceous	Hydrophyte	In rivers, lakes, oxbow lakes
P. pusillus L.	Perennial herbaceous	Hydrophyte	In rivers, lakes
Stuckenia pectinata (L.) Börner	Perennial herbaceous	Hydrophyte	In fresh water lakes
**Melanthiaceae**			
Paris quadrifolia L.	Perennial herbaceous	Hygrophyte	Through excessively damp birch groves
Veratrum lobelianum Bernh.	Perennial herbaceous	Hygrophyte	In flood meadows, damp grassy valleys of rivers, streams, damp intermountain depressions
V. nigrum L.	Perennial herbaceous	Xerophyte	Along the steppe, dry, gravelly slopes
**Liliaceae**			
Fritillaria meleagroides Patrin ex Schult. & Schult.f.	Perennial herbaceous	Xeromesophyte	In the steppes, across damp meadows
F. verticillata Willd.	Perennial herbaceous	Xerophyte	On dry rocky slopes
Gagea bulbifera (Pall.) Salisb.	Perennial herbaceous	Mesoxerophyte	On steppe-grazed gravelly slopes, steppe meadows
G. fedtschenkoana Pascher	Perennial herbaceous	Mesoxerophyte	In the north-eastern foothills of the city of Koktau, in caragana communities (*Caragana frutex* (L.) C. Koch)
G. filiformis (Ledeb.) Kar. & Kir.	Perennial herbaceous	Mesoxerophyte	In steppe meadows, slopes, wormwood-shrub communities
G. fragifera (Vill.) E. Bayer & G. López	Perennial herbaceous	Hygromesophyte	In the damp meadows
G. granulosa Turcz.	Perennial herbaceous	Mesophyte	In meadows, tracts, among plantings of Populus tremula L.
Lilium martagon L.	Perennial herbaceous	Mesophyte	Along forested and grassy slopes, forest edges
Tulipa altaica Pall. ex Spreng.	Perennial herbaceous	Xerophyte	In the steppes, less often on rocky slopes
T. sylvestris subsp. *australis* (Link) Pamp.	Perennial herbaceous	Xerophyte	In the steppes, on rocky slopes
T. uniflora (L.) Besser ex Baker	Perennial herbaceous	Xeropetrophyte	On rocky mountain steppes, less often on rocky slopes, pebble deposits

**Orchidaceae**			
Dactylorhiza fuchsii (Druce) Soó	Perennial herbaceous	Mesophyte	In birch groves, on moderately moist, humus-rich soils
D. incarnata (L.) Soó	Perennial herbaceous	Hygrophyte	In damp marshy meadows, along the banks of reservoirs
D. maculata (L.) Soó	Perennial herbaceous	Hygrophyte	In marshy meadows, on the outskirts of swamps
D. majalis (Rchb.) P.F. Hunt & Summerh.	Perennial herbaceous	Hygrophyte	Through damp and swampy meadows, river banks
D. majalis subsp. *baltica* (Klinge) H.Sund.	Perennial herbaceous	Hygrophyte	In damp meadows, lowland swamps
D. viridis (L.) R. M. Bateman, Pridgeon & M. W. Chase	Perennial herbaceous	Mesophyte	In sparse forests, on forest edges and clearings
Epipactis helleborine (L.) Crantz	Perennial herbaceous	Mesophyte	In sparse deciduous forests, along forest edges, forest glades
Orchis militaris L.	Perennial herbaceous	Mesophyte	In forest clearings, meadows
**Iridaceae**			
Iris ludwigii Maxim.	Perennial herbaceous	Xeromesophyte	On gravelly slopes, steppe meadows, in sparse pine forests.
I. glaucescens Bunge	Perennial herbaceous	Xeromesophyte	On rocky slopes, gravelly steppes
I. halophila Pall.	Perennial herbaceous	Xeromesophyte	In dry saline meadows, along river valleys
I. bloudowii Ledeb.	Perennial herbaceous	Xeromesophyte	Along the mountain steppe slopes
I. ruthenica Ker Gawl.	Perennial herbaceous	Xeromesophyte	In steppe meadows, mountain pine forests, meadow slopes
**Asphodelaceae**			
Eremurus altaicus (Pall.) Steven	Perennial herbaceous	Xerophyte	In dry steppes, on dry stony slopes
**Amaryllidaceae**			
Allium altaicum Pall.	Perennial herbaceous	Petrophyte	Along sparse mountain pine forests, gravelly ridge tops, rocky south-eastern and south-western slopes, along mattress-like granitoids
A. ubsicola Regel	Perennial herbaceous	Mesoxerophyte	On the rocky steppes, foothill slopes
A. angulosum L.	Perennial herbaceous	Mesophyte	On the floodplain rocky meadows
A. caeruleum Pall.	Perennial herbaceous	Xeromesophyte	In the steppe meadows, in the ravines in the bushes, near the rocks
A. flavescens Besser	Perennial herbaceous	Xeromesophyte	In the cereal steppes, on rocky slopes
A. flavidum Ledeb.	Perennial herbaceous	Mesophyte	In forested and rocky meadows, less often, bushy north-western slopes
A. hymenorhizum Ledeb.	Perennial herbaceous	Hygrophyte	In excessively moistened alkaline meadows, grassy swamps, and moist shrub communities
A. lineare L.	Perennial herbaceous	Xeromesophyte	Along the dry foothill steppes, steppe-like, sparsely bushed slopes
A. nutans L.	Perennial herbaceous	Xeromesophyte	In foothill steppes, on rocky and gravelly slopes, rocky outcrops, xerophilous mountain pine forests

A. pallasii Murray	Perennial herbaceous	Mesoxerophyte	In the deserted steppes, on the gravelly slopes of the foothills
A. rubens Spreng.	Perennial herbaceous	Mesophyte	Along rock cracks, rocky slopes, and mountain pine forests
A. saxatile M. Bieb.	Perennial herbaceous	Petrophyte	On the rocky mountain slopes
A. schoenoprasum L.	Perennial herbaceous	Mesophyte	In damp forb-cereal, sedge meadows, less often, in sparse plantings of Salix cinerea L
A. strictum Schrad.	Perennial herbaceous	Petrophyte	On rocky slopes, foothill steppes, ravines
A. tulipifolium Ledeb.	Perennial herbaceous	Mesopetrophyte	On the gravelly slopes of the low mountains
**Asparagaceae**			
Asparagus officinalis L.	Perennial herbaceous	Xeromesophyte	In steppe and floodplain meadows, among thickets of bushes
Convallaria majalis L.	Perennial herbaceous	Mesophyte	In deep shady gullies in poplar-willow groves
**Typhaceae**			
Typha angustifolia L.	Perennial herbaceous	Hygrophyte	Along the banks of rivers, lakes, swamps
T. latifolia L.	Perennial herbaceous	Hygrophyte	Along the banks of rivers, lakes and swamps
T. laxmannii Lepech.	Perennial herbaceous	Hygrophyte	Along the swampy, damp shores of lakes, on the outskirts of marshes
**Juncaceae**			
Juncus atratus Krock.	Perennial herbaceous	Hygrophyte	Through swampy areas, river banks and lakes
J. bufonius L.	Annual herbaceous	Hygrophyte	In coastal damp places
J. compressus Jacq.	Perennial herbaceous	Hygrophyte	In excessively wet areas, along floodplain and marshy meadows, river banks, and lakes
J. gerardi Loisel.	Perennial herbaceous	Hygrophyte	On the damp coastal meadows
J. ranarius Songeon & EPPerrier	Annual herbaceous	Hygrophyte	On damp sandy places, coastal sands
J. salsuginosus Turcz. ex CAMey.	Perennial herbaceous	Hygrophyte	Along the alkaline meadows and salted banks of reservoirs
Luzula multiflora (Ehrh.) Lej.	Perennial herbaceous	Mesophyte	On moderately moist forest slopes
**Cyperaceae**			
Carex acuta L.	Perennial herbaceous	Hygrophyte	Along the clay banks of rivers, along the damp coastal lawns
C. arnellii Christ ex Scheutz	Perennial herbaceous	Mesophyte	In small-leaved forests, on forest edges, among bushes
C. atherodes Spreng.	Perennial herbaceous	Hygrophyte	In damp floodplain meadows and swamps
C. bohemica Schreb.	Perennial herbaceous	Hygrophyte	Along the damp slopes with mixed grasses, coastal meadows of lakes, damp meadows
C. cespitosa L.	Perennial herbaceous	Hygrophyte	In swamps, damp meadows

C. curaica Kunth	Perennial herbaceous	Hygrophyte	Along the sandy banks of rivers, in floodplain meadows
C. elongata L.	Perennial herbaceous	Hygrophyte	In forest meadows, along the banks of streams and rivers
C. humilis Leyss.	Perennial herbaceous	Mesoxerophyte	On gravelly mountain slopes, mountain steppes, rocky pine forests
C. microglochin Wahlenb.	Perennial herbaceous	Hygrophyte	In marshy meadows and lawns, along the banks of rivers and streams
C. oederi var. Oederi	Perennial herbaceous	Hygrophyte	On damp sandy river banks, wet meadows
C. pallescens L.	Perennial herbaceous	Mesophyte	Through grassy forests, forest glades, forest edges
C. pamirensis CBClarke	Perennial herbaceous	Hygrophyte	In swamps, meadows, along the banks of rivers, streams, in the coastal zone of lakes
C. panicea L.	Perennial herbaceous	Hygrophyte	Through swamps and damp meadows
C. pediformis CA Mey.	Perennial herbaceous	Mesopetrophyte	In mountain steppes, forest-steppes, on dry rocky slopes
C. pediformis var. *macroura* (Meinsh.) Kük.	Perennial herbaceous	Mesoxerophyte	In dry, light coniferous and mixed forests, on forest edges, and gravelly slopes
C. praecox Schreb.	Perennial herbaceous	Mesoxerophyte	In steppe-like dry meadows, sandy slopes, steppes, sparse pine and small-leaved forests
C. pseudocyperus L.	Perennial herbaceous	Hygrophyte	Along the banks of reservoirs, in the coastal zone of lakes
C. songarica Schrenk	Perennial herbaceous	Hygrophyte	In marshy meadows, along the banks of rivers and streams, lakes, damp pebbles, from plains to the middle mountain zone
C. supina Willd. ex Wahlenb.	Perennial herbaceous	Mesoxerophyte	In dry steppes, mountain pine forests
C. turkestanica Regel	Perennial herbaceous	Xerophyte	In the steppes, fine-grained rocky slopes, desert steppes
C. utriculata Boott	Perennial herbaceous	Hygrophyte	Along the banks of rivers, lakes, grassy swamps
C. vulpina L.	Perennial herbaceous	Hygrophyte	Through swamps, damp meadows, and river banks
C. vesicaria L.	Perennial herbaceous	Hygrophyte	In sedge bogs, marshy meadows, along the banks of rivers and lakes
Cyperus fuscus L.	Annual herbaceous	Hygrophyte	In damp meadows, along muddy and sandy banks of reservoirs
C. glomeratus L.	Annual herbaceous	Hygrophyte	In damp meadows, along the banks of reservoirs, on silty-sandy soil
Isolepis setacea (L.) R.Br.	Annual herbaceous	Hygrophyte	Along the sandy banks of reservoirs
Schoenoplectiella supina (L.) Lye	Annual herbaceous	Hygrophyte	Along the muddy sandy banks of rivers and lakes
Schoenoplectus lacustris (L.) Palla	Perennial herbaceous	Hygrophyte	Along the banks of reservoirs, in grassy swamps, grassy meadows
Scirpus sylvaticus L.	Perennial herbaceous	Hygrophyte	In damp meadows, along swampy banks
S. tabernaemontani (CCGmel.) Palla	Perennial herbaceous	Hygrophyte	Along the shores of lakes, rivers, and swamps
Trichophorum pumilum (Vahl) Schinz & Thell.	Perennial herbaceous	Hygrophyte	In saline and marshy meadows, along sandy river banks
**Poaceae**			

Agropyron cristatum (L.) Gaertn.	Perennial herbaceous	Xeromesophyte	On rocky steppe slopes, dry meadows, in thickets of bushes
A. desertorum (Fisch. ex Link) Schult.	Perennial herbaceous	Mesopetrophyte	In the rocky steppes up to the lower mountain belt
Agroelymus hajastanica (Tzvelev) Chepinoga	Perennial herbaceous	Mesophyte	In steppe-grass meadows
Agrostis gigantea Roth .	Perennial herbaceous	Hygromesophyte	Through meadows, riverside sands, pebbles, forest edges
Alopecurus pratensis L .	Perennial herbaceous	Mesohygrophyte	In damp meadows, saline meadows
Anthoxanthum nitens (Weber) Y. Schouten & Veldkamp	Perennial herbaceous	Xerophyte	Through the steppe meadows
Avenula pubescens (Huds.) Dumort.	Perennial herbaceous	Mesophyte	In meadows, forest edges, in bushes
Brachypodium pinnatum (L.) P. Beauv.	Perennial herbaceous	Xeromesophyte	In dry forests, steppe shrubs, edges of shrub thickets
Bromus inermis Leyss.	Perennial herbaceous	Xeromesophyte	In steppe meadows, meadow slopes, in bushes
Calamagrostis epigejos (L.) Roth	Perennial herbaceous	Mesoxerophyte	On foothill plains, in intermountain depressions, dry river valleys
Campeiostachys dahurica (Turcz. ex Griseb.) BRBaum, JLYang & C.Yen	Perennial herbaceous	Mesophyte	Along rocky mountain slopes, steppe foothill meadows, river pebbles
Cleistogenes squarrosa (Trin.) Keng	Perennial herbaceous	Xerophyte	Along the steppe slopes of the low mountains
Dactylis glomerata L.	Perennial herbaceous	Mesophyte	Through floodplain and dry meadows, edges of aspen and birch forests, bushes
Deschampsia cespitosa (L.) P.Beauv.	Perennial herbaceous	Mesohygrophyte	In meadows, along the shores of lakes, swamps
Echinochloa crus-galli (L.) P.Beauv.	Annual herbaceous	Mesophyte	Like weeds, near housing
Elymus caninus (L.) L.	Perennial herbaceous	Mesophyte	In aspen forests, mesophilic shrub communities
E. confusus (Roshev.) Tzvelev	Perennial herbaceous	Mesophyte	In coastal forb-grass meadows, ravines in aspen plantations, river pebbles, rocky mountain meadows, in steppe shrub communities
E. dentatus (Hook.f.) Tzvelev	Perennial herbaceous	Xeromesophyte	Along the steppe mountain meadows, stony, bushy slopes
E. gmelinii (Trin.) Tzvelev	Perennial herbaceous	Xeromesophyte	Along dry rocky slopes, sparsely bushed cereal steppes
E. mutabilis (Drobow) Tzvelev	Perennial herbaceous	Mesoxerophyte	Through sparse aspen forests, mesophilic shrubby slopes, coastal meadows with mixed grasses, and river pebbles
E. repens (L.) Gould	Perennial herbaceous	Xeromesophyte	In the steppes, meadows, fallow lands, fixed sands, river valleys, bush thickets, in sparse forest communities
E. sibinicus Kotukhov	Perennial herbaceous	Mesophyte	Along the coastal meadows with mixed grasses and shrubs
Festuca altissima All.	Perennial herbaceous	Mesophyte	In shady mixed forests, shady gorges
F. valesiaca Schleich. ex Gaudin	Perennial herbaceous	Xerophyte	In dry steppes, rocky slopes
Helictochloa hookeri (Scribn.) Romero Zarco	Perennial herbaceous	Xerophyte	On dry steppes, mountain slopes
Helictotrichon desertorum (Less.) Pilg	Perennial herbaceous	Xerophyte	On the rocky steppes

Hordeum brevisubulatum (Trin.) Link	Perennial herbaceous	Hygromesophyte	Along river valleys, moderately damp meadows, salted meadows
H. jubatum L.	Perennial herbaceous	Mesophyte	Like weeds near a house
Leymus angustus (Trin.) Pilg.	Perennial herbaceous	Xeromesophyte	In deserted steppes, saline meadows, along dry river valleys
L. chinensis (Trin.) Tzvelev	Perennial herbaceous	Mesophyte	On shaded dry lawns
L. paboanus (Claus) Pilg.	Perennial herbaceous	Xerophyte	In saline meadows and steppes
L. secalinus (Georgi) Tzvelev	Perennial herbaceous	Xeromesophyte	In coastal sands, mountain pine forests
Lolium giganteum (L.) Darbysh.	Perennial herbaceous	Mesophyte	In forests and bushes, in damp meadows
L. pratense (Huds.) Darbysh.	Perennial herbaceous	Mesophyte	In damp meadows, birch and aspen groves
Melica altissima L.	Perennial herbaceous	Xeromesophyte	In thickets of bushes and their edges
M. transsilvanica Schur	Perennial herbaceous	Xeromesophyte	Along the steppe rocky slopes, forest edges and thickets of bushes
M. nutans L.	Perennial herbaceous	Mesophyte	In deciduous forests, forest clearings
Milium effusum L.	Perennial herbaceous	Mesophyte	In forest plantations
Neotrinia splendens (Trin.) M.Nobis, P.D. Gudkova & A.Nowak	Perennial herbaceous	Xerophyte	In deserts, dry steppes
Phalaris arundinacea L.	Perennial herbaceous	Hygrophyte	Along the banks of rivers, lakes, streams, in damp meadows
Phleum pratense L.	Perennial herbaceous	Mesophyte	Through foothill meadows, birch and aspen groves
P. phleoides (L.) H.Karst.	Perennial herbaceous	Xeromesophyte	On dry meadows, steppes, rocky slopes
Phragmites australis (Cav.) Trin. ex Steud.	Perennial herbaceous	Hygrophyte	Along the banks of rivers, lakes, in flood meadows, damp depressions
Piptatherum songaricum (Trin. & Rupr.) Roshev.	Perennial herbaceous	Xerophyte	On rocky slopes, cliffs, up to the middle mountain zone
Poa alpigena Lindm.	Perennial herbaceous	Hygromesophyte	Along the banks of streams, rivers, and less often on gravelly slopes
P. angustifolia L.	Perennial herbaceous	Xeromesophyte	On dry meadows, bushy slopes
P. pratensis L.	Perennial herbaceous	Mesophyte	In meadows, forest clearings, along river valleys, among bushes
P. remota Forselles	Perennial herbaceous	Hygrophyte	Through swampy forests, forest glades, and stream banks
P. sibirica Roshev.	Perennial herbaceous	Mesophyte	In meadows, among bushes, in mountain pine forests
P. annua L.	Annual herbaceous	Mesophyte	Along roads, on lawns, coastal pebbles, near houses
P. palustris L.	Perennial herbaceous	Hygromesophyte	In meadows, meadow swamps, excessively damp forest glades, coastal sands and pebbles, among bushes
P. versicolor Besser	Perennial herbaceous	Xeromesophyte	On dry mixed-grass meadows, meadow steppes, outskirts of pine forests, floodplain pebbles
Psathyrostachys juncea (Fisch.) Nevski	Perennial herbaceous	Xerophyte	In dry steppes, on steppe rocky slopes

Rostraria cristata (L.) Tzvelev	Perennial herbaceous	Xeromesophyte	On dry mixed-grass meadows, meadow steppes, outskirts of pine forests, floodplain pebbles
Setaria viridis (L.) P.Beauv.	Annual herbaceous	Mesophyte	Like weeds, along roadsides, dry gravelly slopes
Stipa borysthenica Klokov ex Prokudin	Perennial herbaceous	Xerophyte	On eroded clays, fine-grained steppes
S. capillata L.	Perennial herbaceous	Mesopetrophyte	In foothill steppes, steppe meadows, rocky and steppe slopes
S. glareosa PA Smirn.	Perennial herbaceous	Xerophyte	On the rocky and gravelly slopes of the lower belt
S. lessingiana Trin. & Rupr.	Perennial herbaceous	Xerophyte	On poorly formed substrates, on cereal steppes, fine-grass slopes
S. orientalis Trin.	Perennial herbaceous	Xerophyte	On gravelly slopes, desertified clay-gravelly areas
S. pennata L.	Perennial herbaceous	Xerophyte	On steppe rocky slopes, steppe meadows of the lower and middle mountain belts
S. sareptana AK Becker	Perennial herbaceous	Xerophyte	On open gravelly slopes, steppe meadows
S. zalesskii Wilensky	Perennial herbaceous	Xerophyte	Through steppe grass meadows.
**Papaveraceae**			
Chelidonium majus L.	Perennial herbaceous	Mesophyte	In shady places, bushes, along river banks, near housing, as a weed
Corydalis nobilis (L.) Pers.	Perennial herbaceous	Xeromesophyte	In gorges, rocky slopes, bushes
Fumaria schleicheri Soy.-Will.	Annual herbaceous	Mesoxerophyte	On rocky slopes, cliffs and, as a weed, in crops, often near housing
Hypecoum pendulum var. *pendulum*	Annual herbaceous	Xeromesophyte	On gravelly, rocky, clayey slopes of the foothills, often as a weed
**Berberidaceae**			
Berberis sibirica Pall.	Bush	Petrophyte	On rocks, rocky slopes
Gymnospermium altaicum (Pall.) Spach	Perennial herbaceous	Mesoxerophyte	Along the mountain slopes, aspen-fir forests
**Ranunculaceae**			
Aconitum anthora subsp. *anthora*	Perennial herbaceous	Xeromesophyte	In steppe meadows, in thickets of bushes, among meadow grasses
A. septentrionale Koelle	Perennial herbaceous	Mesophyte	In forests, on forest edges, forest clearings, along river valleys
A. volubile Pall. ex Koelle	Perennial herbaceous	Hygrophyte	In forests, along their edges, river valleys, swamp edges, lake shores
Adonis villosa Ledeb.	Perennial herbaceous	Xerophyte	In the mountain steppes
A. volgensis Steven ex DC.	Perennial herbaceous	Mesoxerophyte	In fescue and feather grass steppes, along the edges of bushes
Anemone caerulea DC.	Perennial herbaceous	Mesophyte	In forests, bushes, on lawns
A. sylvestris L.	Perennial herbaceous	Xeromesophyte	In dry steppe meadows, edges of bushes
Caltha palustris L.	Perennial herbaceous	Hygrophyte	In damp meadows, on the outskirts of swamps, on the banks of streams and rivers

Clematis alpina subsp. *sibirica* (L.) Kuntze	Perennial herbaceous	Mesophyte	Along the forest edges
C. glauca Willd.	Perennial herbaceous	Mesophyte	In river valleys, poplar and willow floodplain forests, in the lower part of mountain slopes
C. integrifolia L.	Perennial herbaceous	Mesoxerophyte	Among the shrubs in steppe and floodplain meadows, steppe slopes
C. orientalis L.	Perennial herbaceous	Mesoxerophyte	On dry foothill slopes, in ravines, and less often in rocky pine forests
C. songorica Bunge	Perennial herbaceous	Mesoxerophyte	On rocky scree and slopes, in meadows in the lower mountain belt
Delphinium cyananthum Nevski	Perennial herbaceous	Xerophyte	On steppe slopes, steppe meadows
D. dictyocarpum DC.	Perennial herbaceous	Xeromesophyte	In steppe and dry meadows, in birch groves, in thickets of bushes
D. elatum L.	Perennial herbaceous	Mesophyte	Through birch and aspen groves, deep shady gorges
Pulsatilla patens (L.) Mill.	Perennial herbaceous	Xeromesophyte	In dry steppes on mountain slopes, sparse pine forests
Ranunculus acris L.	Perennial herbaceous	Hygrophyte	In flood meadows, marshy places, in forest groves
R. auricomus L.	Perennial herbaceous	Mesophyte	In flood meadows, on the edges of small-leaved forests
R. cassubicus L.	Perennial herbaceous	Mesophyte	In birch and aspen groves
R. gmelinii DC.	Perennial herbaceous	Hygrophyte	On the damp banks of rivers, streams, in swamps
R. grandifolius CAMey.	Perennial herbaceous	Hygromesophyte	In damp meadows, forest meadows, forest groves, along rivers
R. minimus (L.) EHLKrause	Annual herbaceous	Mesophyte	In crops, on pastures, as a weed
R. monophyllus Ovcz.	Perennial herbaceous	Hygromesophyte	Through damp places, forest groves, among meadow grasses
R. pedatifidus Sm.	Perennial herbaceous	Xerophyte	On dry meadows, less often, saline meadows
R. polyanthemos L.	Perennial herbaceous	Xerophyte	In dry meadows, in steppe depressions, in pine plantations
R. polyphyllus Waldst. & Kit. ex Willd.	Perennial herbaceous	Hygrophyte	In damp depressions, swamps
R. polyrhizos Stephan ex Willd.	Perennial herbaceous	Xeromesophyte	In dry meadows, fallow lands, rocky slopes, in sparse bushes
R. repens L.	Perennial herbaceous	Hygrophyte	In damp meadows, in river valleys, along muddy river banks
R. testiculatus Crantz	Annual herbaceous	Xerophyte	In the steppes, on dry slopes, on desolate pastures
Thalictrum aquilegiifolium var. *sibiricum* Regel & Tiling	Perennial herbaceous	Hygromesophyte	In riverside meadows, in sparse groves, shrub thickets
T. foetidum L.	Perennial herbaceous	Xerophyte	In rocky and gravelly places
T. flavum L.	Perennial herbaceous	Hygromesophyte	Along river valleys, thickets of bushes, edges of forest groves, edges of grassy swamps
T. isopyroides CAMey.	Perennial herbaceous	Xerophyte	In the rocky steppes, on the cliffs
T. minus L.	Perennial herbaceous	Mesoxerophyte	In steppe meadows, in thickets of bushes

T. simplex L.	Perennial herbaceous	Mesophyte	Along river valleys, floodplain meadows, bush thickets, and, less commonly, birch-aspen groves
Trollius altaicus CAMey.	Perennial herbaceous	Hygromesophyte	In forest meadows, along river valleys
**Paeoniaceae**			
Paeonia anomala L.	Perennial herbaceous	Mesophyte	In the forests, in forest meadows, in foothill clearings
P. tenuifolia L.	Perennial herbaceous	Xerophyte	On the steppe slopes, open rocky slopes of the lower belt
**Grossulariaceae**			
Ribes aciculare Sm.	Bush	Petrophyte	On open rocky slopes
R. atropurpureum CA Mey.	Bush	Mesophyte	In the shady forest groves
R. nigrum L.	Bush	Mesophyte	Through damp forests, rocky shaded cliffs
R. saxatile Pall.	Subshrub	Petrophyte	On rocks, scree, rocky slopes, destroyed cliffs
**Saxifragaceae**			
Saxifraga sibirica L.	Perennial herbaceous	Petrophyte	In fir stands, along cracks and rock ledges
**Crassulaceae**			
Hylotelephium telephium (L.) H.Ohba	Perennial herbaceous	Xeromesophyte	In forest and floodplain meadows, sparse rocky pine forests, steppe rocky meadows
Orostachys spinosa (L.) Sweet	Perennial herbaceous	Xerophyte	In the steppe zone on open rocky and gravelly slopes
Phedimus hybridus (L.) 't Hart	Perennial herbaceous	Petrophyte	In mountain steppes, on rocky substrates, rocky outcrops
Sedum alberti Regel	Perennial herbaceous	Petrophyte	On rocky and gravelly mountain slopes
**Fabaceae**			
Astragalus agrestis Douglas	Perennial herbaceous	Xeromesophyte	In meadows, less often on gravelly slopes of low mountains, in steppes
A. alpinus L.	Perennial herbaceous	Xerophyte	On rocky slopes, gravel beds, less often, meadows, in pine sparse forests
A. buchtormensis Pall.	Perennial herbaceous	Xeromesophyte	On steppe meadows, rocky slopes
A. chaetolobus Bunge	Subshrub	Petrophyte	On the southern, rocky slopes of low mountains and foothill plains
A. danicus Retz.	Perennial herbaceous	Mesophyte	In forest clearings, floodplain and steppe meadows
A. kaufmannii Krylov	Perennial herbaceous	Xerophyte	On gravel-gravel areas, mixed-grass steppe meadows
A. onobrychis L.	Perennial herbaceous	Xeromesophyte	Across the steppe grass-forb meadows, foothill plains
A. puberulus Ledeb.	Perennial herbaceous	Xerophyte	Along the dry mountain slopes, rocky steppes
A. scabrisetus Bong.	Perennial herbaceous	Petrophyte	Along rocky slopes, clay outcrops
A. schanginianus Pall.	Perennial herbaceous	Xeromesophyte	On the steppe rocky slopes of the mountains, in the thickets of bushes

A. sulcatus L.	Perennial herbaceous	Xeromesophyte	In steppe meadows, in river floodplains
A. syriacus L.	Subshrub	Xerophyte	On open rocky slopes of low mountains, less often, steppe meadows
A. testiculatus Pall.	Perennial herbaceous	Xerophyte	Along rocky slopes, steppe meadows
Caragana arborescens Lam.	Bush	Mesophyte	Along river banks, sparse forests, forest edges and clearings, rocky slopes
C. frutex (L.) K.Koch	Bush	Xerophyte	In the steppes, on dry gravelly slopes, dry ravines, edges of mountain forests
C. spinosa (L.) Vahl ex Hornem.	Bush	Petrophyte	Along mountain steppes, rocky slopes, upland rocky pine forests
Glycyrrhiza glabra L.	Subshrub	Xerophyte	In dry steppes, rocky semi-deserts
G. uralensis Fisch. ex DC.	Perennial herbaceous	Xerophyte	In saline meadows, dry rocky steppes
Hedysarum gmelinii Ledeb.	Perennial herbaceous	Petrophyte	On rocky outcrops, gravel, rubble and steppe slopes
H. splendens Fisch. ex DC.	Perennial herbaceous	Xerophyte	On dry rocky slopes, steppe meadows
Lathyrus frondosus (Freyn) Kuprian.	Perennial herbaceous	Xerophyte	In saline meadows:
L. gmelinii (Fisch. ex Ser.) Fritsch	Perennial herbaceous	Hygromesophyte	In forest clearings, along the bottoms of tracts, river valleys
L. humilis (Ser.) Fisch. ex Spreng.	Perennial herbaceous	Mesoxerophyte	In birch-aspen groves, pine plantations, on rocky slopes
L. pisiformis L.	Perennial herbaceous	Mesophyte	In meadows, in forests, among bushes
L. vernus (L.) Bernh.	Perennial herbaceous	Mesophyte	In shady places, aspen-birch groves, bushes
Lotus angustissimus L.	Annual herbaceous	Xeromesophyte	In damp saline meadows, in river valleys
Medicago falcata L.	Perennial herbaceous	Xeromesophyte	On dry floodplain meadows, grassy and gravelly slopes, forest edges
M. lupulina L.	Annual/biennial herbaceous	Mesophyte	In meadows, forest edges, wastelands
M. platycarpos (L.) Trautv.	Perennial herbaceous	Xerophyte	Along rocky dry slopes, steppe meadows
Melilotus albus Medik.	Annual/biennial herbaceous	Mesophyte	In the steppe meadows, like weeds, along the roadsides, in fallow lands
M. officinalis (L.) Lam.	Biennial herbaceous	Mesophyte	In the steppe meadows, on the edges of roads, on fallow lands
Onobrychis arenaria (Kit.) DC.	Perennial herbaceous	Xerophyte	In the steppes, on dry meadows, on the southern and south-eastern slopes of the foothills, on fallow lands
Oxytropis floribunda (Pall.) DC.	Perennial herbaceous	Xeromesophyte	In the steppes, along sandy and pebble river banks
O. pilosa (L.) DC.	Perennial herbaceous	Xeromesophyte	In steppe meadows, gravelly, shrub-forb meadows
O. songorica (Pall.) DC	Perennial herbaceous	Petrophyte	On rocky, gravelly slopes
O. sulphurea (Fisch. ex DC.) Ledeb.	Perennial herbaceous	Mesophyte	In the mountain meadows
Sophora alopecuroides L.	Perennial herbaceous	Mesophyte	In river valleys, saline meadows, fallow lands, and wastelands

Trifolium fragiferum L.	Perennial herbaceous	Mesophyte	In wet saline meadows
T. hybridum L.	Perennial herbaceous	Hygromesophyte	Through damp meadows, river valleys
T. lupinaster L.	Perennial herbaceous	Xeromesophyte	On dry meadows, gravelly slopes, forest meadows, meadow steppes, sparse rock pine forests
T. pratense L.	Perennial herbaceous	Mesophyte	In sparse meadows, fallow lands: found throughout the entire territory
T. repens L.	Perennial herbaceous	Mesophyte	In drylands, floodplain meadows, fallow lands, along roads
Trigonella grandiflora Bunge	Annual herbaceous	Xeromesophyte	On fallow lands, steppe dry meadows, as a weed
Vicia cracca L.	Perennial herbaceous	Mesophyte	In bushes, meadows, in the lower mountain belt
V. megalotropis Ledeb.	Perennial herbaceous	Mesophyte	On the edges of birch-aspen groves, meadows, in river valleys and light pine forests
V. sepium L.	Perennial herbaceous	Mesophyte	On the slopes and bottoms of gorges, meadows, along river floodplains, meadow clearings, forest edges, thickets of bushes
V. sylvatica L.	Perennial herbaceous	Mesophyte	In deciduous and pine forests, on the slopes of forest ravines
V. tenuifolia Roth	Perennial herbaceous	Xeromesophyte	In dry meadows, forest edges, forest clearings, mixed-grass steppes, in the lower mountain belt
**Polygalaceae**			
Polygala comosa Schkuhr	Perennial herbaceous	Xeromesophyte	Across meadow steppes, rocky mountain slopes, and pine forests
P. sibirica L.	Perennial herbaceous	Xerophyte	On dry rocky outcrops
**Rosaceae**			
Agrimonia eupatoria subsp. *asiatica* (Juz.) Skalický	Annual/biennial herbaceous	Xeromesophyte	On mountain slopes, in river floodplains, aspen-birch groves, along the edges of bushes
Alchemilla murbeckiana Buser	Perennial herbaceous	Hygromesophyte	On the banks of rivers and in birch groves
A. sibirica Zämelis	Perennial herbaceous	Mesophyte	In meadows, forest edges and clearings, along deep gorges
Argentina anserina (L.) Rydb.	Perennial herbaceous	Hygromesophyte	In coastal damp places, wet lawns
Coluria geoides (Pall.) Bunge	Perennial herbaceous	Petrophyte	On mountain steppes, rocky slopes
Cotoneaster laxiflorus J. Jacq. ex Lindl.	Bush	Xeromesophyte	On rocky slopes, cliffs, in shrub communities
C. multiflorus Bunge	Bush	Petrophyte	On rocks, gravelly and stony mountain slopes
C. uniflorus Bunge	Subshrub	Petrophyte	On rocky slopes, placers, cliffs
C. oliganthus Pojark.	Bush	Petrophyte	On the rocky mountain slopes
Crataegus chlorocarpa Lenné & K. Koch	Tree	Mesophyte	In the shrub thickets of the lower mountain belt
C. sanguinea Pall	Bush	Xeromesophyte	On the slopes of gorges, gullies, along river valleys

Dasiphora fruticosa L.	Subshrub	Hygrophyte	Along river banks, flood meadows, river valleys
D. parvifolia (Fisch. ex Lehm.) Juz.	Subshrub	Petrophyte	In the steppes, rocky pine forests, on rocks, mattress-like granitoids
Farinopsis salesoviana (Stephan)	Subshrub	Hygromesophyte	On rocky and gravelly slopes, along river banks, in sparse rocky pine plantations
Filipendula ulmaria (L.) Maxim.	Perennial herbaceous	Hygromesophyte	In river floodplains, near streams, in damp places, tall-grass meadows
F. vulgaris Moench	Perennial herbaceous	Xeromesophyte	In steppe habitats, dry meadows, in bushes
Fragaria viridis Weston	Perennial herbaceous	Mesophyte	In aspen-birch groves, on grassy slopes, forest edges, clearings, steppe meadows
Geum rivale L.	Perennial herbaceous	Hygrophyte	In damp meadows, along river banks, on the outskirts of swamps
G. urbanum L.	Perennial herbaceous	Hygrophyte	In damp grassy meadows, along the banks of rivers and streams
Malus baccata (L.) Borkh.	Tree	Petrophyte	In the gorges
Potentilla acaulis L.	Perennial herbaceous	Petrophyte	On the steppe gravelly slopes and rocky peaks of the hills
P. argentea L.	Perennial herbaceous	Mesoxerophyte	On steppe meadows, forest edges, along fallow lands
P. asiatica (Th.Wolf) Juz.	Perennial herbaceous	Mesophyte	In mountain meadows with mixed grasses, river floodplains
P. chrysantha Trevir.	Perennial herbaceous	Mesophyte	In meadows, in sparse birch-aspen groves, on mountain grassy slopes
P. humifusa Willd. ex DFK Schltdl.	Perennial herbaceous	Xerophyte	On steppe meadows, rocky-gravelly slopes
P. inclinata Vill.	Perennial herbaceous	Xerophyte	In the steppes, on steppe meadows
P. multifida L.	Perennial herbaceous	Petrophyte	On rocky and gravelly slopes, steppe meadows
P. pedata Willd. ex Hornem.	Perennial herbaceous	Xerophyte	On the fine-earth-gravelly slopes of the foothills
P. soongorica Bunge	Perennial herbaceous	Xerophyte	On dry steppe and gravelly slopes of low mountains
P. supina L.	Annual herbaceous	Hygromesophyte	In damp meadows, coastal lawns
P. tanacetifolia Willd. ex DFK Schltdl.	Perennial herbaceous	Mesoxerophyte	On rocky open slopes, forest glades, in the steppes
P. virgata Lehm.	Perennial herbaceous	Xerophyte	In the steppe, on steppe meadows
Prunus fruticosa Pall.	Bush	Xeromesophyte	In the steppe thickets of bushes, tracts
P. padus L.	Tree	Hygromesophyte	In river valleys, streams, ravines, ravines, in shrub thickets: found throughout the entire territory
P. tenella Batsch	Bush	Xerophyte	On the open south-eastern slopes of the hills and foothills
Sanguisorba officinalis L.	Perennial herbaceous	Hygromesophyte	In damp meadows, grassy slopes, along valleys and river banks
Sibbaldianthe bifurca (L.) Kurtto & T. Erikss.	Subshrub	Xerophyte	On rocky and stony steppes, steppe meadows, stony slopes: found throughout the entire territory.

Sorbus aucuparia subsp. *glabrata* (Wimm. & Grab.) Hedl.	Tree	Mesophyte	On the edges of the pine forest
Spiraea hypericifolia L.	Bush	Xeromesophyte	On rocky slopes, steppe meadows, in thickets of bushes
S. media Schmidt	Bush	Mesophyte	In forest groves, on shady slopes, rocks, sometimes in mixed-grass meadows
S. trilobata L.	Subshrub	Petrophyte	On open steppe rocky slopes, in rocky pine forests
Rosa acicularis Lindl.	Bush	Mesophyte	In forests, on forest and rocky slopes, in thickets of bushes, mountain gorges, rocky pine forests
R. alberti Regel	Bush	Xeromesophyte	In the mountain forest belt, on forest edges, near cliffs, in bushes
R. laxa Retz.	Bush	Xeromesophyte	On mountain slopes, river banks and valleys, forest and shrub slopes (does not form dense thickets)
R. spinosissima L.	Bush	Xerophyte	On the mountain slopes and steppe meadows
Rubus caesius L.	Bush	Hygromesophyte	In forest groves, thickets of bushes, along river banks
R. idaeus L.	Bush	Mesophyte	Along mountain slopes, river banks, forest edges, aspen-birch groves
R. saxatilis L.	Subshrub	Xeromesophyte	In pine forests, in thickets of bushes, on rocky slopes, river valleys
**Elaeagnaceae**			
Hippophae angustifolia L.	Tree	Hygrophyte	Along the banks and in the valleys of dry rivers, an alien species
H. rhamnoides L.	Bush	Hygrophyte	It grows along river banks and pebbles.
**Rhamnaceae**			
Frangula alnus Mill.	Bush	Xeromesophyte	On forest edges, among bushes, in river floodplains, ravines, and gravelly slopes
**Ulmaceae**			
Ulmus pumila L.	Tree	Mesophyte	Along the roadsides, near housing
**Cannabaceae**			
Cannab is sativa L.	Annual herbaceous	Mesophyte	As a weed, in crops, near housing, on roadsides
Humulus lupulus L.	Perennial herbaceous	Mesophyte	Along river valleys, ravines, and bush thickets
**Urticaceae**			
Urtica cannabina L.	Perennial herbaceous	Mesophyte	Rocky slopes, wastelands, roadsides, near housing
U. dioica L.	Perennial herbaceous	Mesophyte	In forests, ravines, river banks, often as a weed near housing
U. urens L.	Annual herbaceous	Mesophyte	In damp places, near houses, in vegetable gardens
**Betulaceae**			
Betula microphylla Bunge	Tree	Xerophyte	Along the steppe river valleys

B. pendula Roth	Tree	Petrophyte	In groves, rocky slopes, along gorges, river valleys
B. pendula subsp. *pendula*	Tree	Xeromesophyte	In the steppe and shrub belts, on gravelly and rocky slopes
B. pubescens Ehrh.	Tree	Hygrophyte	In the gorges of mountain rivers
B. tianschanica Rupr.	Tree	Petrophyte	Along gravelly slopes, valleys of mountain rivers
**Cucurbitaceae**			
Ecballium elaterium (L.) A.Rich.	Annual herbaceous	Hygrophyte	Adventitious, in damp places, in river valleys
**Celastraceae**			
Parnassia palustris L.	Perennial herbaceous	Hygrophyte	In the steppe zone, in damp, less often, swampy meadows, on the banks of rivers and streams
**Hypericaceae**			
Hypericum elongatum Ledeb. ex Rchb.	Perennial herbaceous	Xeropetrophyte	Along dry rocky and gravelly slopes, rocks of eastern exposure
H. hirsutum L.	Perennial herbaceous	Mesoxerophyte	Across tall-grass meadow steppes and wooded mountain slopes, rocky steppes
H. perforatum L.	Perennial herbaceous	Mesophyte	In damp meadows, forest edges, clearings of island forests, rocky slopes, in river valleys, gorges, gullies
**Violaceae**			
Viola arvensis Murray	Annual herbaceous	Mesophyte	In open areas, meadows, fields as a weed
V. canina L.	Perennial herbaceous	Hygromesophyte	Along river banks, forest edges, in small-leaved forests, and bush thickets
V. disjuncta W. Becker	Perennial herbaceous	Mesophyte	In meadows, sparse forests, deep ravines
V. hirta L.	Perennial herbaceous	Xeromesophyte	In forests, bushes, on grassy steppe slopes, tracts
V. macroceras Bunge	Perennial herbaceous	Petrophyte	On mossy rocky outcrops, rocky slopes, in thickets of bushes, gullies and gorges
V. mirabilis L.	Perennial herbaceous	Mesophyte	Along forested slopes, ravines, deep gorges
V. rupestris FW Schmidt	Perennial herbaceous	Xeropetrophyte	On dry grassy and rocky slopes, in rocky pine forests
V. tricolor L.	Annual herbaceous	Mesophyte	In dry meadows, along forest edges
**Salicaceae**			
Salix alba L.	Tree	Hygrophyte	Along the valleys, river banks
S. bebbiana Sarg.	Tree	Hygrophyte	Along river valleys, tracts, bushy mesophilic slopes, along the shores of lakes
S. caprea L.	Tree	Hygrophyte	Along floodplains and river banks, riverside meadows
S. cinerea L.	Tree	Hygrophyte	Along river valleys, springs, lake shores, marshy meadows, hummocky bogs
S. gmelinii Pall	Tree	Hygrophyte	Along the banks of rivers and springs

S. pentandra L.	Tree	Hygrophyte	Along river valleys, swampy meadows with mixed grasses, hummocky bogs
S. pyrolifolia Ledeb	Tree	Hygrophyte	Along the banks of rivers, streams, marshy meadows, hummocky bogs
S. rorida Laksch.	Tree	Hygrophyte	Along the pebble banks of rivers and springs
S. rosmarinifolia L.	Bush	Hygrophyte	Through excessively damp meadows, swamps, marshy willow thickets, hummocky bogs
S. tenuijulis Ledeb.	Tree	Hygrophyte	Along river valleys, on coastal pebbles
S . triandra L.	Tree	Hygrophyte	Along valleys, banks, marshy meadows, and the outskirts of swamps
S. turanica Nasarow	Tree	Hygrophyte	Along the coastal strip of the spring, in the damp lakeside meadows
S. viminalis L.	Tree	Hygrophyte	Along the banks of rivers, lakes, and less often, in marshy meadows and lowlands
Populus laurifolia Ledeb.	Tree	Hygrophyte	Along the river valleys
P. nigra L.	Tree	Hygrophyte	Along the river banks
P. tremula L.	Tree	Mesophyte	In the north, north-west slopes of tracts
**Euphorbiaceae**			
Euphorbia humifusa Willd.	Annual herbaceous	Petrophyte	On the rocks and rocky slopes of the foothills, clay outcrops
E. latifolia Ledeb.	Perennial herbaceous	Mesophyte	In thickets of bushes, on northern gravelly slopes, along the edges of birch-aspen groves
E. macrorhiza Ledeb.	Perennial herbaceous	Petrophyte	On the steppe rocky and gravelly southern and south-eastern slopes
E. microcarpa (Prokh.) Krylov	Perennial herbaceous	Mesophyte	In mixed-grass meadows, in sparse birch-aspen groves, less often in mountain pine forests
E. pilosa L.	Perennial herbaceous	Mesophyte	In birch-aspen groves, forest edges, and mixed-grass meadows
E. subcordata Ledeb.	Perennial herbaceous	Xeromesophyte	In the steppes, on steppe meadows, rocky and gravelly slopes
E. uralensis Fisch. ex Link	Perennial herbaceous	Xeromesophyte	In damp meadows, salty lawns, gravel, shrub edges, and, less commonly, steppe meadows
E. virgata Waldst. & Kit.	Perennial herbaceous	Hygromesophyte	In damp meadows, edges of birch-aspen groves, in sparse bushes, river valleys
**Geraniaceae**			
Erodium stephanianum Willd.	Annual/biennial herbaceous	Mesoxerophyte	On sandy and pebble river banks, dry rocky slopes
Geranium collinum Stephan ex Willd.	Perennial herbaceous	Mesophyte	On plains, meadow slopes, forest clearings, less often, in birch-aspen groves:
G. divaricatum Ehrh.	Annual herbaceous	Mesophyte	In shrubby thickets, on gravelly mountain slopes, often as a weed
G. pratense L.	Perennial herbaceous	Hygromesophyte	In meadows, forest edges, in river floodplains

G. pseudosibiricum J.Mayer	Perennial herbaceous	Hygromesophyte	Through meadows, meadow slopes, gorges, mountain river valleys
G. robertianum L.	Annual/biennial herbaceous	Mesophyte	In shady ravines, gullies, aspen-birch groves
G. sibiricum L.	Perennial herbaceous	Mesophyte	As a weed, near housing on pastures and along roads
G. sylvaticum L.	Perennial herbaceous	Hygromesophyte	In damp meadows, forest edges, forest clearings, in river valleys
**Lythraceae**			
Lythrum nanum Kar. & Kir.	Annual herbaceous	Xerophyte	In saline places
L. tribracteatum Salzm. ex Spreng.	Annual herbaceous	Hygrophyte	Along the outskirts of swamps, river banks
L. virgatum L.	Perennial herbaceous	Hygrophyte	In excessively moist steppe meadows, swamps, and along river banks
**Onagraceae**			
Epilobium angustifolium L.	Perennial herbaceous	Mesophyte	In birch-aspen groves, bushes, on the northwestern slopes
E. hirsutum L.	Perennial herbaceous	Hygrophyte	In grassy swamps, along damp banks of rivers, lakes, streams
E. palustre L.	Perennial herbaceous	Hygrophyte	In flood meadows, grassy swamps, groundwater outlets
**Nitrariaceae**			
Peganum harmala L.	Perennial herbaceous	Xerophyte	In the deserted steppes
**Sapindaceae**			
Acer negundo L.	Tree	Mesophyte	In ravines, gorges, river valleys, near housing
**Rutaceae**			
Dictamnus albus L.	Perennial herbaceous	Xerophyte	In steppe meadows, foothill slopes, among thickets of bushes
**Malvaceae**			
Malva pusilla Sm.	Annual herbaceous	Hygromesophyte	In damp places
M. thuringiaca (L.) Vis.	Perennial herbaceous	Xeromesophyte	In the steppe zone, thickets of bushes, in meadows
M. verticillata L.	Annual herbaceous	Mesophyte	In garbage areas and as a weed in vegetable gardens
**Thymelaeaceae**			
Daphne altaica Pall.	Bush	Mesophyte	In shrub thickets, on the slopes of low mountains
Thymelaea passerina (L.) Coss. & Germ.	Perennial herbaceous	Xeropetrophyte	On rocky slopes, less often as a weed, in crops
**Brassicaceae**			

Alyssum alyssoides (L.) L	Annual herbaceous	Petrophyte	On the rocky, gravelly slopes of the low mountains
A. desertorum Stapf	Annual herbaceous	Xerophyte	On the steppe rocky slopes of low mountains, steppe and rocky outskirts of mountain pine communities
A. lenense Adams	Subshrub	Xerophyte	On the steppe rocky slopes, in mountain pine forests
A. turkestanicum Regel & Schmalh.	Annual herbaceous	Xerophyte	On the dry rocky slopes of the low mountains
Arabidopsis thaliana (L.) Heynh.	Annual herbaceous	Mesoxerophyte	On rocky mountain slopes, steppe meadows, and bush edges
Armoracia rusticana G. Gaertn., B. Mey. & Scherb.	Perennial herbaceous	Mesophyte	Near housing, gardens
Barbarea vulgaris WT Aiton	Biennial herbaceous	Mesophyte	Occasionally on mountain slopes, in meadows, fields, or as a weed
Berteroa incana (L.) DC.	Biennial herbaceous	Xeromesophyte	In steppe meadows, fallow lands, hills and dry slopes, as a weed, in fields, roadsides and housing
Brassarda juncea (L.)	Annual herbaceous	Mesophyte	As a weed, in fallow lands, vegetable gardens, near roads
Brassica rapa L.	Annual/biennial herbaceous	Mesophyte	As a weed, in fields, gardens, near houses and roads
Bunias orientalis L.	Perennial herbaceous	Mesophyte	As a weed, on fallow lands, roadsides, fields
Camelina microcarpa Andrz. ex DC.	Annual/biennial herbaceous	Mesophyte	Like weeds, near roads, houses, in fields
Capsella bursa-pastoris (L.) Medik.	Annual/biennial herbaceous	Mesophyte	Everywhere like a weed
Cardamine impatiens L.	Annual/biennial herbaceous	Hygromesophyte	In birch and aspen groves, along the banks of rivers and streams
Chorispora bungeana Fisch. & CA May.	Perennial herbaceous	Xeromesophyte	On the rocky north-western slopes of low mountains, rarely, as a weed, near housing
C. sibirica (L.) DC.	Annual herbaceous	Xeromesophyte	On steppe meadows, less often on rocky slopes and weeds near housing
C. tenella (Pall.) DC.	Annual herbaceous	Xerophyte	On the deserted rocky slopes of the low mountains
Clausia aprica (Stephan ex Willd.)	Perennial herbaceous	Xeromesophyte	In steppe meadows, rocky and gravelly slopes, in sparse upland pine forests
Crucihimalaya mollissima (CAMey.) Al-Shehbaz, O'Kane & RAPrice	Perennial herbaceous	Xerophyte	On rocks, stony and gravelly mountain slopes
Descurainia sophia (L.) Webb ex Prantl	Annual/biennial herbaceous	Mesophyte	Near housing, less often, on the slopes of low mountains
Draba cana Rydb.	Subshrub	Hygromesophyte	On gravelly and rocky slopes, along the banks of streams
D. nemorosa L.	Annual herbaceous	Xeromesophyte	In steppe meadows, forest clearings, floodplain steppe meadows, in shady gorges, sparse forests
D. ochroleuca Bunge	Subshrub	Hygromesophyte	Along the banks of rivers, shady gorges, shady mountain meadows
D. sibirica (Pall.) Thell.	Subshrub	Mesophyte	On lawns, coastal meadows

Erysimum altaicum CA Mey	Biennial herbaceous	Xerophyte	In mountain steppes, on steppe meadows, on southern gravelly slopes
E. diffusum Ehrh.	Biennial herbaceous	Xerophyte	In rocky steppes, pine forests, dry meadows, dry rocky meadows
E. odoratum Ehrh.	Biennial herbaceous	Mesoxerophyte	In the steppes, on rocky slopes, fallow lands, like weeds
E. vassilczenkoi Polatschek	Biennial herbaceous	Petrophyte	On the rocky mountain slopes
Galitzkya spathulata (Stephan ex Willd.)	Perennial herbaceous	Xerophyte	On rocks, stony and gravelly slopes of mountains, hills, and hills
Hesperis sibirica L.	Biennial herbaceous	Hygromesophyte	Through birch and aspen groves, river banks, and bushes
H. sibirica subsp. *pseudonivea* (Tzvelev)	Biennial herbaceous	Mesophyte	In forests, meadows, rocky slopes, in bushes
Isatis costata CA Mey.	Biennial herbaceous	Xerophyte	In the steppes, on rocky and gravelly slopes
Lepidium cordatum Willd. ex Steven	Perennial herbaceous	Mesoxerophyte	On salted lawns
L. draba L.	Perennial herbaceous	Mesoxerophyte	In clay steppes, often as a weed, in fields and along roadsides
L. latifolium L.	Perennial herbaceous	Xeromesophyte	As a weed, near housing, in dry riverbeds, less often, on saline lawns
L. perfoliatum L.	Annual/biennial herbaceous	Xerophyte	On steppe meadows, clayey salty lawns
L. ruderale L.	Annual/biennial herbaceous	Mesophyte	As a weed, near houses, in vacant lots, along roads
Neslia paniculata (L.) Desv.	Annual herbaceous	Mesoxerophyte	Like a weed, in fields, mountain slopes, bushy lowlands, near housing
Noccaea perfoliata (L.) Al-Shehbaz	Annual herbaceous	Xerophyte	On dry rocky and gravelly slopes, dry meadows
Odontarrhena obovata CAMey.	Subshrub	Petrophyte	On fine-grained slopes
O. tortuosa (Willd.)	Subshrub	Xerophyte	On the rocky steppe slopes
Parrya stenocarpa Kar. & Kir.	Perennial herbaceous	Mesoxerophyte	On steppe meadows, rocks, gravelly and rocky mountain slopes
Rhammatophyllum fruticulosum (CAMey.)	Subshrub	Petrophyte	On granite mountain slopes, rocky slopes
Rorippa palustris (L.) Besser	Annual herbaceous	Hygrophyte	In swamps, river banks, lakes, garbage dumps
Sisymbrium loeselii L.	Annual herbaceous	Mesoxerophyte	On steppe meadows, meadow and gravelly slopes
S. polymorphum (Murray) Roth	Perennial herbaceous	Xerophyte	On steppe meadows, gravelly and rocky slopes
Sterigmostemum caspicum (Lam.) Kuntze	Perennial herbaceous	Petrophyte	In clayey-saline rocky places
Stevenia cheiranthoides DC.	Perennial herbaceous	Xerophyte	On mountain steppes, rocky and gravelly slopes
Thlaspi arvense L.	Annual herbaceous	Xeromesophyte	On rocks, as a weed, near dwellings, in crops, along forest edges, less often, in river valleys
Thlaspi ceratocarpon (Pall.) Murray	Annual herbaceous	Xerophyte	On dry meadows, along the trails of steppe mountains
Turritis glabra L.	Biennial herbaceous	Xeromesophyte	In steppe meadows, rocky slopes, in dry rocky pine forests, weeds near dwellings

**Santalaceae**			
Thesium refractum CA Mey	Perennial herbaceous	Mesoxerophyte	Across the flat steppes, forest edges, and mountain slopes
T. repens Ledeb.	Perennial herbaceous	Mesophyte	Along river valleys, steppe meadows, forest edges
**Plumbaginaceae**			
Goniolimon speciosum (L.) Boiss.	Perennial herbaceous	Xerophyte	In the steppes, on the gravelly slopes of the low mountains
**Polygonaceae**			
Atraphaxis frutescens (L.) K. Koch	Subshrub	Xerophyte	On the rocky slopes of the foothills
Fallopia convolvulus (L.) Á. Love	Annual herbaceous	Mesoxerophyte	In fields, fallow lands, scree slopes, among bushes
Koenigia alpina (All.) TM Schust. & Reveal	Perennial herbaceous	Mesophyte	On moist meadow mountain slopes, forest edges
Persicaria hydropiper (L.) Delarbre	Annual herbaceous	Hygrophyte	Along the banks of rivers, in damp meadows
Polygonum aviculare L.	Annual herbaceous	Mesophyte	Along the roadsides, near housing, in vacant lots
Polygonum patulum M. Bieb.	Annual herbaceous	Mesophyte	In the feather grass-wormwood steppes, like a weed, along the roads
Rheum compactum L.	Perennial herbaceous	Xerophyte	On rocky and gravelly steppe slopes
Rumex acetosa L.	Perennial herbaceous	Mesophyte	In meadows, in sparse forests, on grassy slopes
R. aquaticus L.	Perennial herbaceous	Hygrophyte	Along the banks of rivers and lakes, in marshy meadows
R. confertus Willd	Perennial herbaceous	Mesophyte	In meadows, on slopes, forest clearings, like a weed, in pastures
R. crispus L.	Perennial herbaceous	Mesophyte	In meadows, on the edges of fields, sometimes as a weed
R. marschallianus Rchb.	Annual herbaceous	Hygrophyte	Along valleys and river banks, occasionally in damp places
R. thyrsiflorus Fingerh.	Perennial herbaceous	Hygrophyte	In flood meadows, along river valleys, on pebbles
**Caryophyllaceae**			
Agrostemma githago L.	Annual herbaceous	Xeromesophyte	In fallow lands, along roads, in crops
Cerastium arvense L.	Perennial herbaceous	Xeromesophyte	In dry meadows, dry rocky and gravelly slopes, in sparse tree communities
C. davuricum Fisch. ex Spreng.	Perennial herbaceous	Mesophyte	In the forest belt in meadows, forest edges, in thickets of bushes
C. falcatum (Gren.) Bunge ex Fenzl	Perennial herbaceous	Mesophyte	In sparse birch-aspen groves, mountain river valleys, shady gorges
C. pauciflorum Steven ex Ser.	Perennial herbaceous	Mesophyte	In sparse coniferous and mixed forests
Dianthus chinensis L.	Perennial herbaceous	Xerophyte	In pine forests, steppes, steppe meadows, rocky and gravelly slopes
D. leptopetalus Willd.	Perennial herbaceous	Xerophyte	In the steppes, on steppe meadows, dry slopes
D. ramosissimus Pall. ex Poir.	Perennial herbaceous	Xeromesophyte	In meadow steppes, pine forests

D. recticaulis Ledeb.	Annual/biennial herbaceous	Xerophyte	On rocky and gravelly slopes, in sparse pine forests
D. superbus L.	Perennial herbaceous	Mesophyte	In dry meadows, along the forest edges of birch groves
Gypsophila altissima L.	Perennial herbaceous	Xerophyte	Across steppe meadows, rocky slopes, and sparse pine forests
G. paniculata L.	Perennial herbaceous	Xerophyte	On steppe meadows, gravelly slopes, along the edges of sparse pine plantations
Herniaria polygama J.Gay	Annual herbaceous	Xerophyte	In the steppes, dry pine forests
Holosteum umbellatum subsp. *glutinosum* (M.Bieb.) Nyman	Annual herbaceous	Xerophyte	Along dry steppe and rocky slopes
Mesostemma dichotomum (L.) Arabi, Rabeler & Zarre	Perennial herbaceous	Xerophyte	On dry and gravelly slopes
M. amblyosepalum (Schrenk) Chepinoga	Perennial herbaceous	Xerophyte	On dry and rocky slopes
Moehringia trinervia (L.) Clairv.	Annual herbaceous	Mesophyte	In shady and sparse forests, among bushes
M. lateriflora (L.) Fenzl	Perennial herbaceous	Mesophyte	In sparse aspen-birch groves, pine forests, and bushes
Sabulina regeliana (Trautv.) Dillenb. & Kadereit	Annual herbaceous	Xerophyte	In saline areas and steppe slopes
S. litwinowii (Schischk.) Dillenb. & Kadereit	Perennial herbaceous	Xerophyte	On rocks, alluvial pebbles
Saponaria officinalis L.	Perennial herbaceous	Mesophyte	Along river valleys, in forests and meadows, less often as a weed
Silene altaica Pers.	Perennial herbaceous	Xerophyte	On gravelly slopes, rocky outcrops
S. chalcedonica (L.) EHL Krause	Perennial herbaceous	Mesophyte	In meadows, in shrub thickets, along river valleys
S. chlorantha (Willd.) Ehrh.	Perennial herbaceous	Xeromesophyte	In the steppes, steppe meadows, pine forests, and along their edges
S. incurvifolia Kar. & Kir.	Perennial herbaceous	Xerophyte	On rocky and gravelly slopes
S. latifolia subsp. *alba* (Mill.) Greuter & Burdet	Perennial herbaceous	Mesophyte	In the meadows, like a weed, in crops
S. nutans L.	Perennial herbaceous	Mesophyte	In sparse forests, damp meadows
S. sibirica (L.) Pers.	Perennial herbaceous	Xeromesophyte	In the steppes, sparse birch groves
S. viscosa (L.) Pers.	Perennial herbaceous	Xerophyte	On rocky slopes, in the steppes
S. vulgaris (Moench) Garcke	Perennial herbaceous	Mesophyte	In meadows, in sparse forests
S. wolgensis (Hornem.) Otth	Perennial herbaceous	Xeromesophyte	In the steppes, steppe meadows, sparse aspen forests, on grassy slopes
Stellaria bungeana Fenzl	Perennial herbaceous	Mesophyte	In shady forests, on grassy slopes, deep shady gorges and ravines
S. crassifolia Ehrh.	Perennial herbaceous	Hygrophyte	In swamps, wet meadows
S. graminea L.	Perennial herbaceous	Mesophyte	In meadows, forest edges, in thickets of bushes, river valleys
S. longifolia Muhl. ex Willd.	Perennial herbaceous	Hygrophyte	In damp shady places and along river banks

S. media (L.) Vill.	Annual/biennial herbaceous	Mesophyte	As a weed, in vegetable gardens, near houses, along roadsides, forest edges, and gravel banks
S. neglecta (Lej.) Weihe	Annual/biennial herbaceous	Mesophyte	Through weedy places
**Amaranthaceae**			
Amaranthus retroflexus L	Annual herbaceous	Mesophyte	As a weed, in crops, vegetable gardens, along roads and houses
Atriplex tatarica L.	Annual herbaceous	Mesophyte	In wastelands, vegetable gardens
Axyris amaranthoides L.	Annual herbaceous	Xerophyte	In wastelands, in crops, on fallow lands, along roads, in garbage dumps, and less often on rocky mountain slopes
A. hybrida L.	Annual herbaceous	Mesophyte	In weedy places, on fallow lands, on rocky and gravelly slopes
Bassia prostrata (L.) Beck	Subshrub	Xerophyte	On the rocky and gravelly slopes of the low mountains
Blitum virgatum L.	Annual herbaceous	Mesoxerophyte	On rocky-gravelly slopes, pebbles, less often, as a weed
Camphorosma songorica Bunge	Annual herbaceous	Xerophyte	In damp places in the steppe zone
Ceratocarpus arenarius L.	Annual herbaceous	Mesophyte	Through weedy places
Chenopodium album L.	Annual herbaceous	Mesophyte	As a weed, in fields, gardens, along roads
C. karoi (Murr) *Aellen*	Annual herbaceous	Mesophyte	In vegetable gardens, weedy places
Dysphania botrys (L.) Mosyakin & Clemants	Annual herbaceous	Mesoxerophyte	On sandy soils, rocky slopes, dry riverbeds, and weedy places
Krascheninnikovia ceratoides (L.) Gueldenst.	Perennial herbaceous	Xerophyte	Across the steppes, rocky and gravelly slopes
Oxybasis rubra (L.) S.Fuentes, Uotila & Borsch	Annual herbaceous	Mesophyte	Near housing, along the roadsides
Teloxys aristata (L.) Moq.	Annual herbaceous	Mesoxerophyte	On rocky outcrops and, like weeds, in fields, along roads
**Portulacaceae**			
Portulaca oleracea L.	Annual herbaceous	Mesophyte	Across fields, vegetable gardens, wastelands
**Polemoniaceae**			
Polemonium caeruleum L.	Perennial herbaceous	Mesophyte	In low mountains, valley meadows, along river valleys, and at the edges of bush thickets
**Primulaceae**			
Androsace filiformis Retz.	Annual herbaceous	Mesophyte	Through forest glades, floodplain damp meadows
A. maxima L.	Annual herbaceous	Xerophyte	In the steppes, on steppe meadows, rocky and gravelly slopes
A. septentrionalis L.	Annual/biennial herbaceous	Mesopetrophyte	In mountain pine forests, meadows, rocky slopes
Lysimachia vulgaris L.	Perennial herbaceous	Hygrophyte	Across damp meadows, river banks, lakes

Primula longiscapa Ledeb.	Perennial herbaceous	Hygromesophyte	In the steppe zone, in damp and flooded meadows, along river valleys
P. nutans Georgi	Perennial herbaceous	Mesophyte	In mountain pine forests, meadows, rocky slopes
P. veris subsp. *macrocalyx* (Bunge) Lüdi	Perennial herbaceous	Mesophyte	In floodplain meadows, in sparse aspen forests, in ravines among bushes
**Rubiaceae**			
Galium aparine L.	Annual herbaceous	Mesophyte	As a weed, in crops, on roadsides, near housing
G. boreale L.	Perennial herbaceous	Mesophyte	In meadows, in bushes, on mountain slopes, river valleys, sparse forests
G. humifusum M. Bieb.	Perennial herbaceous	Mesophyte	On rocks, among bushes, in river floodplains
G. palustre L.	Perennial herbaceous	Hygrophyte	On the outskirts of swamps, in willow thickets, in excessively wet meadows
G. rubioides L.	Perennial herbaceous	Mesophyte	In mixed-grass meadows, forest slopes, along river valleys, in bush thickets
G. spurium L.	Annual herbaceous	Mesophyte	In the bushes
G. verum L.	Perennial herbaceous	Mesoxerophyte	In the steppes, on steppe-like mountain meadows, steppe-like mountain slopes
G. verum subsp. *verum*	Perennial herbaceous	Xerophyte	In steppe meadows, steppes, rocky slopes, river banks, mountain pine forests
**Gentianaceae**			
Gentiana decumbens Lf	Perennial herbaceous	Mesophyte	In mixed-grass meadows, along river valleys, gorges
Swertia dichotoma L.	Annual herbaceous	Petrophyte	On the gravelly slopes of the foothills
**Boraginaceae**			
Cerinthe minor L.	Annual/perennial herbaceous	Mesophyte	In fields, along roads, on shrubby slopes
Cynoglossum officinale L.	Biennial herbaceous	Mesophyte	As a weed, in vacant lots, near houses, along roads
C. viridiflorum Pall. ex Lehm.	Perennial herbaceous	Petrophyte	On rocky slopes, in thickets of bushes and foothills
Echium vulgare L.	Annual/biennial herbaceous	Mesophyte	In wastelands, fallow lands, and along roads
Eritrichium pauciflorum DC.	Subshrub	Petrophyte	On rocks, stony and gravelly mountain slopes, closed gravel beds
Lappula intermedia (Ledeb.) Popov	Annual herbaceous	Xerophyte	In the gravelly steppes, on gravelly slopes
L. patula (Lehm.) Menyh.	Annual herbaceous	Xerophyte	Through the deserted steppe areas
L. rupestris (Schrenk) Gürke	Annual/biennial herbaceous	Petrophyte	Along the rocky lowlands
L. squarrosa (Retz.) Dumort.	Annual/biennial herbaceous	Petrophyte	Along rocky slopes, trails of low mountains, foothills
L. stricta (Ledeb.) Gürke	Annual herbaceous	Xerophyte	On rocky and gravelly slopes, in desert steppes
L. tenuis (Ledeb.) Gürke	Annual herbaceous	Xerophyte	In the dry steppes, on the rocky slopes of the foothills

Lithospermum officinale L.	Perennial herbaceous	Xeromesophyte	In dry meadows, shrub thickets, island forests
Myosotis imitata Serg.	Perennial herbaceous	Mesophyte	Through forest meadows and forests, steppe mountain meadows
M. krylovii Serg.	Perennial herbaceous	Mesophyte	In birch and aspen groves, forest meadows with mixed grasses
M. scorpioides subsp. *scorpioides*	Perennial herbaceous	Hygrophyte	In damp forest meadows, swampy coastal areas of the floodplain
M. sparsiflora JC Mikan ex Pohl	Annual herbaceous	Mesophyte	On rocky and weedy places, wet lawns
M. stricta Link ex Roem. & Schult.	Annual herbaceous	Xerophyte	On dry sandy and gravelly slopes of southern and south-eastern exposures
Nonea pulla (L.) DC	Annual/perennial herbaceous	Xerophyte	In the steppes, rocky steppe meadows
Onosma gmelinii Ledeb.	Perennial herbaceous	Petrophyte	Along rocky slopes and cliffs
O. simplicissima L.	Perennial herbaceous	Petrophyte	On rocky and gravelly slopes, less often on cliffs
Pulmonaria mollis JF Wolff ex Hornem.	Perennial herbaceous	Mesophyte	Along the edges of forest groves, forest tracts, forest river valleys
**Convolvulaceae**			
Calystegia sepium (L.) R.Br.	Perennial herbaceous	Mesophyte	Along ravines, river valleys, in thickets of trees and bushes
Convolvulus arvensis L.	Perennial herbaceous	Mesophyte	In steppe meadows, along river valleys, sparse rocky pine forests, as a weed along roadsides, in crops, on fallow lands
Cuscuta europaea L.	Annual herbaceous	Mesophyte	Parasitizes on herbaceous plants and shrubs
C. lupuliformis Krock.	Annual herbaceous	Mesophyte	Parasitizes on shrubs
**Solanaceae**			
Hyoscyamus niger L.	Annual/biennial herbaceous	Mesophyte	Near housing, on abandoned fields
Physochlaina physaloides (L.) G.Don	Perennial herbaceous	Petrophyte	On open steppe rocky slopes of low mountains, hills, cliffs
Solanum dulcamara L.	Bush	Hygrophyte	In damp thickets of bushes, along the banks of rivers and springs
S. nigrum L.	Annual/perennial herbaceous	Mesophyte	As a weed, in vegetable gardens, near housing, garbage dumps
**Oleaceae**			
Syringa vulgaris L.	Bush	Mesophyte	Through shady ravines and gorges
**Plantaginaceae**			
Linaria acutiloba Fisch.	Perennial herbaceous	Mesophyte	In steppe meadows, forest meadows, in river valleys
L. altaica Fisch.	Perennial herbaceous	Petrophyte	On the rocky and gravelly slopes of the mountain
L. bungei Kuprian.	Perennial herbaceous	Petrophyte	On gravelly and rocky mountain slopes, through dry pine forests
L. hepatica Bunge	Perennial herbaceous	Petrophyte	On the rocky and gravelly slopes of the low mountains

L. vulgaris Mill.	Perennial herbaceous	Petrophyte	In pine forests, on rocky mountain slopes
Plantago lanceolata L.	Perennial herbaceous	Xeromesophyte	On steppe meadows, along river valleys, open dry mountain slopes
P. major L.	Annual/perennial herbaceous	Hygromesophyte	In damp meadows, along river valleys, near roads and houses
P. media L.	Perennial herbaceous	Hygromesophyte	In meadows, along river banks, meadow slopes, forest clearings
P. urvillei Opiz	Perennial herbaceous	Xeromesophyte	In the steppes, dry meadows, river valleys
Veronica anagallis-aquatica L.	Annual herbaceous	Hygrophyte	In excessively damp meadows, along the banks of rivers and streams
V. beccabunga L.	Subshrub	Hygrophyte	Along the banks of rivers, streams and small flowing springs
V. dillenii Crantz	Annual herbaceous	Xerophyte	In the steppes, pine forests of the lowlands, on the steppe slopes
V. krylovii Schischk.	Perennial herbaceous	Xeromesophyte	In dry meadows, in sparse coniferous and deciduous forests
V. longifolia L.	Perennial herbaceous	Hygromesophyte	In damp, mixed-grass meadows, in sparse pine stands, aspen and birch groves
V. pinnata L.	Perennial herbaceous	Xerophyte	On the steppe slopes in the lower mountain belt
V. porphyriana Pavlov	Perennial herbaceous	Mesophyte	In mixed-grass meadows, forest clearings
V. serpyllifolia L.	Perennial herbaceous	Hygromesophyte	On damp places of mixed-grass slopes
V. spicata L.	Perennial herbaceous	Mesoxerophyte	On dry steppe and forest meadows, rocky pine plantations, rocky and steppe slopes
V. spuria L.	Perennial herbaceous	Mesophyte	In the meadow steppes
V. verna L.	Annual herbaceous	Xerophyte	In dry meadows, on rocky slopes, in weedy places
**Scrophulariaceae**			
Scrophularia altaica Murray	Perennial herbaceous	Petrophyte	Along the shady cliffs, rocky river banks
S. nodosa L.	Perennial herbaceous	Hygromesophyte	In overly damp meadows with mixed grass, along the banks of rivers, springs, ravines, and in sparse forests
Verbascum phoeniceum L.	Perennial herbaceous	Xerophyte	In the steppes, on the steppe slopes
V. songaricum Schrenk ex Fisch. & CA May.	Biennial herbaceous	Mesoxerophyte	In dry meadows, sepian and rocky slopes, among bushes
V. thapsus L.	Biennial herbaceous	Mesophyte	Across steppe meadows, rocky slopes, and river valleys
**Lamiaceae**			
Dracocephalum discolor Bunge	Perennial herbaceous	Petrophyte	On open, fine-grained slopes
D. nutans L.	Perennial herbaceous	Mesophyte	In bush thickets, pastures
D. origanoides Stephan ex Willd.	Perennial herbaceous	Petrophyte	On gravelly and rocky slopes
D. peregrinum L.	Perennial herbaceous	Petrophyte	On rocky and gravelly slopes, cliffs

D. ruyschiana L.	Perennial herbaceous	Petrophyte	On rocky meadows, in sparse rocky pine forests
D. thymiflorum L.	Annual herbaceous	Mesophyte	In rocky pine forests, in wastelands, near roads
Galeopsis ladanum L.	Annual herbaceous	Mesophyte	In crops, fallow lands, along roads, sparse pine forests, near housing
G. speciosa Mill.	Annual herbaceous	Mesophyte	In rocky pine forests, in fields, vegetable gardens, near roads and houses
Glechoma hederacea L.	Perennial herbaceous	Hygromesophyte	In damp places, in deciduous shady forests, along river banks
Lagochilus bungei Benth.	Perennial herbaceous	Petrophyte	On rocky and gravelly slopes
Lamium album L.	Perennial herbaceous	Mesophyte	In deciduous forests, shrubs
L. turkestanicum VI Krecz. & Kuprian.	Perennial herbaceous	Mesophyte	In the forest belt of the mountains, in shady forests, along the banks of rivers
Leonurus glaucescens Bunge	Biennial/perennial herbaceous	Petrophyte	On the mountain slopes
Mentha arvensis L.	Perennial herbaceous	Hygrophyte	In the forest belt along wet floodplains and sedge bogs
M. longifolia var. *asiatica* (Boriss.) Rech.f.	Perennial herbaceous	Hygrophyte	Along river valleys, damp floodplain meadows, streams and springs
Nepeta nuda L.	Perennial herbaceous	Xerophyte	On steppe meadows, open slopes
N. schrenkii (Levin) Sennikov	Perennial herbaceous	Petrophyte	In shady gorges, on granites, among rocks, rocky scree
Origanum vulgare L.	Perennial herbaceous	Xerophyte	On dry and steppe meadows, rocky slopes
Phlomoides agraria (Bunge) Adylov, Kamelin & Makhm.	Perennial herbaceous	Mesoxerophyte	Across the gravelly steppes and among sparse bushes
P. oreophila (Kar. & Kir.) Adylov, Kamelin & Makhm.	Perennial herbaceous	Petrophyte	Along the rocky meadow slopes
P. tuberosa (L.) *Moench*	Perennial herbaceous	Xerophyte	In the multi-grass steppes, along the rocky foothills
Prunella vulgaris L.	Perennial herbaceous	Mesophyte	In meadows, forest clearings, in river valleys
Salvia dumetorum Andrz. ex Besser	Perennial herbaceous	Xerophyte	In the steppes, dry steppe meadows
Scutellaria altaica Ledeb. ex Sweet	Subshrub	Xerophyte	On rocky and meadow mountain slopes, steppe meadows
S. galericulata L.	Perennial herbaceous	Hygromesophyte	In the meadows, on damp river banks
S. sieversii Bunge	Perennial herbaceous	Petrophyte	On dry, stony mountain slopes
Stachys palustris L.	Perennial herbaceous	Hygromesophyte	In wet meadows, in river valleys
Thymus altaicus Klokov & Des.-Shost.	Subshrub	Xerophyte	Across rocky mountain steppes, sand deposits, and river banks
T. mongolicus (Ronniger) Ronniger	Subshrub	Petrophyte	On rocky slopes, cliffs, scree
T. pannonicus All.	Subshrub	Xerophyte	Along the steppe slopes of mountains and foothills, steppe meadows, sparse pine forests
T. roseus Schipcz.	Subshrub	Petrophyte	On rocky slopes, low mountains, cliffs, scree

Ziziphora clinopodioides Lam.	Subshrub	Petrophyte	On rocky and gravelly slopes
**Mazaceae**			
Dodartia orientalis L.	Perennial herbaceous	Xerophyte	In the steppes, rocky slopes, steppe meadows
**Orobanchaceae**			
Euphrasia pectinata Ten.	Annual herbaceous	Xerophyte	In the steppes, steppe meadows, mountain slopes
E. × *vernalis* List	Annual herbaceous	Mesophyte	In sparse forests, thickets of bushes, fallow lands, meadows
Odontites vulgaris Moench	Annual herbaceous	Hygrophyte	In damp places, in river floodplains, less often in meadows
Orobanche coerulescens Stephan	Annual/perennial herbaceous	Xerophyte	In the steppes, on rocky slopes, it parasitizes on species of wormwood
O. sordida CA Mey.	Annual/perennial herbaceous	Petrophyte	On rocky slopes, parasitizes *Spiraea hypericifolia* L.
Pedicularis achilleifolia Stephan ex Willd.	Perennial herbaceous	Xerophyte	Along the rocky steppe slopes
P. cystopteridifolia Rydb.	Perennial herbaceous	Hygromesophyte	Along meadow slopes, river valleys
P. physocalyx Bunge	Perennial herbaceous	Xerophyte	In the mountain steppes, on rocky slopes
P. proboscidea Steven	Perennial herbaceous	Mesophyte	In mountain meadows, less often in river valleys
Rhinanthus borbasii subsp. *songaricus* (Sterneck) Soó	Annual herbaceous	Hygromesophyte	In meadows, meadow slopes, along river valleys
R. minor L.	Annual herbaceous	Hygromesophyte	In dry meadows, river banks and lakes
**Campanulaceae**			
Adenophora liliifolia (L.) A.DC.	Perennial herbaceous	Mesophyte	In forest meadows, on meadow mountain slopes, in rocky pine forests
Campanula bononiensis L.	Perennial herbaceous	Xeromesophyte	In steppe meadows, in bushes
C. cervicaria L.	Biennial/perennial herbaceous	Mesophyte	In meadows, on meadow slopes, in sparse forests
C. glomerata L.	Perennial herbaceous	Mesophyte	In meadows, on meadow slopes, in sparse forests, on their edges
C. rapunculoides L.	Perennial herbaceous	Mesophyte	In bushes, on fallow lands, often near housing (possibly an alien species)
C. sibirica L.	Biennial/perennial herbaceous	Xerophyte	In dry meadows and steppes
C. stevenii subsp. *altaica* (Ledeb.) Fed.	Perennial herbaceous	Xerophyte	In steppe meadows, foothill mixed-grass meadows
**Asteraceae**			
Achillea asiatica Serg.	Perennial herbaceous	Xeromesophyte	In meadows, open mountain slopes, in thickets of bushes, steppes
A. millefolium subsp. *millefolium*	Perennial herbaceous	Petrophyte	On the rocky slopes and cliffs, the ridges of the foothills

A. millefolium L.	Perennial herbaceous	Mesoxerophyte	In forest-steppe, steppe zones, in meadows, steppe and meadow mountain slopes
A. salicifolia Besser	Perennial herbaceous	Hygromesophyte	Along the banks of rivers, lakes, meadows, birch groves
Alfredia cernua Cass.	Perennial herbaceous	Mesophyte	Through meadows, coniferous and deciduous forests, and mountain river valleys
Antennaria dioica (L.) Gaertn.	Perennial herbaceous	Xerophyte	In open rocky areas, on dry and open slopes, dry meadows
Arctium tomentosum Mill.	Biennial herbaceous	Mesophyte	Along the roads, like weeds, in the gardens
Artemisia abrotanum L.	Subshrub	Xerophyte	In the steppe zone, damp ravines, birch groves, on rocky slopes
A. absinthium L.	Perennial herbaceous	Mesophyte	As a weed, near roads, housing, fallow lands, pastures
A. annua L.	Annual herbaceous	Mesophyte	Near housing, along the foothill valleys
A. armeniaca Lam.	Perennial herbaceous	Xeromesophyte	In the steppes, steppe meadows, rocky slopes, in thickets of steppe shrubs
A. austriaca Jacq.	Perennial herbaceous	Xeromesophyte	In steppe meadows, pine forests, river valleys
A. rupestris L.	Perennial herbaceous	Xerophyte	In the steppe zone, rocky riverbeds, gravelly slopes
A. sericea (Besser) Weber ex Stechm.	Perennial herbaceous	Mesoxerophyte	In steppe meadows, in thickets of bushes, birch and aspen groves, in gravelly and rocky places
A. atifolia Ledeb.	Perennial herbaceous	Xeromesophyte	In steppe meadows, feather-grass and mixed-grass steppes, edges of pine forests, less often, gravelly and rocky slopes
A. campestris L.	Perennial herbaceous	Xerophyte	In steppe meadows, pine forests, steppes, terrace slopes
A. dracunculus L.	Perennial herbaceous	Xeromesophyte	On steppe slopes, birch groves, sparse pine stands, in thickets of steppe shrubs
A. glauca Pall. ex Willd.	Perennial herbaceous	Xeromesophyte	In steppe meadows, sparse birch forests, meadows, river valleys, and less commonly fallow lands
A. juncea Kar. & Kir.	Subshrub	Xerophyte	In the steppe zone, on the gravelly and rocky slopes of hills
A. laciniata Willd.	Perennial herbaceous	Mesophyte	Among the bushes
A. marschalliana Spreng.	Perennial herbaceous	Xerophyte	In steppe meadows, in rocky pine forests, on rocky and gravelly slopes
A. pontica L.	Perennial herbaceous	Xeromesophyte	In meadow steppes, on steppe meadows, in birch-aspen groves, thickets of bushes
A. pubescens var. *pubescens*	Perennial herbaceous	Xerophyte	In sparse birch groves, pine forests, on rocky arches, rocky and gravelly slopes of low mountains
A. scoparia Waldst. & Kit.	Annual/perennial herbaceous	Xerophyte	On steppe meadows, in pine forests, on rocky and gravelly slopes, like weeds in the fields
A. sieversiana Ehrh. ex Willd.	Annual/biennial herbaceous	Xerophyte	On steppe meadows, rocky mountain slopes, as a weed along the roadsides, near housing
A. sublessingiana (B.Keller) Krasch. ex Poljakov	Subshrub	Petrophyte	On the gravelly foothills and slopes of low mountains

A. vulgaris L.	Perennial herbaceous	Mesophyte	In forests, bushes, forest edges, along river valleys, on mountain slopes, fallow lands, as a weed near housing
Aster alpinus L	Perennial herbaceous	Petrophyte	On rocky and steppe mountain slopes, rocky outcrops
A. altaicus Willd.	Perennial herbaceous	Xerophyte	On steppe meadows, rocky and gravelly slopes
Bidens tripartita L.	Annual herbaceous	Hygrophyte	In the swamps, along the damp banks of rivers
Carduus crispus L.	Perennial herbaceous	Mesophyte	In wastelands, bushes, forest edges, near roads and houses
C. nutans L.	Biennial herbaceous	Mesophyte	In damp places, pastures, along roads, on rocky slopes
Centaurea cyanus L.	Annual herbaceous	Mesophyte	On fallow lands, as a weed near housing, in cereal crops
C. scabiosa L.	Perennial herbaceous	Xeromesophyte	In meadows, among bushes, sparse rock pine forests
Cichorium intybus L.	Perennial herbaceous	Mesophyte	In meadows, river floodplains, often as a weed, along roads
Cirsium arvense var. *vestitum* Wimm. & Grab.	Perennial herbaceous	Mesophyte	In the valleys of steppe rivers, like weeds in crops
C. esculentum (Siev.) CAMey.	Perennial herbaceous	Xeromesophyte	In mixed-grass damp meadows, in steppes, low-grass meadows
C. helenioides (L.) Hill	Perennial herbaceous	Mesophyte	Along meadow-forb slopes, fir forests, river valleys
C. serratuloides (L.) Hill	Perennial herbaceous	Mesophyte	Through sparse coniferous and deciduous forests, river valleys
C. vulgare (Savi) Ten.	Biennial herbaceous	Mesophyte	In meadows, fallow lands, wastelands, rocky places
Chondrilla aspera Poir.	Perennial herbaceous	Xerophyte	In the steppes, on rocky and gravelly slopes, cliffs, along river valleys
C. brevirostris Fisch. & CA May.	Perennial herbaceous	Mesoxerophyte	In the steppe zone, pine plantations, steppe meadows, less often as a weed
C. canescens Kar. & Kir.	Perennial herbaceous	Petrophyte	On rocky and gravelly slopes of low mountains, pebbles
C. rouillieri Kar. & Kir.	Perennial herbaceous	Mesophyte	In pine plantations, less often as a weed in fields
Cota tinctoria (L.) J.Gay	Biennial/perennial herbaceous	Xeromesophyte	In dry places, disturbed areas, fields, as weeds near housing
Crepidiastrum tenuifolium (Willd.) Sennikov	Perennial herbaceous	Xerophyte	Along the rocks, rocky steppes
Crepis lyrata (L.) Froel.	Perennial herbaceous	Mesophyte	Through sparse forests, forest glades
C. tectorum L.	Annual herbaceous	Mesophyte	In meadows, fields, fallow lands, along river valleys
Erigeron acris L.	Biennial/perennial herbaceous	Mesophyte	In meadows, fallow lands, in pine forests
E. acris subsp. *podolicus* (Besser) Nyman	Biennial/perennial herbaceous	Mesophyte	In meadows, birch groves, fallow lands
E. canadensis L.	Annual herbaceous	Xeromesophyte	As a weed, in crops, fallow lands, near housing, rarely in steppe forests and rocky plantations of Scots pine (Pinus sylvestris L.)
E. lonchophyllus Hook.	Perennial herbaceous	Mesophyte	In the foothill plains, in the meadows, along the Rivers

E. politus Fr.	Biennial/perennial herbaceous	Xeromesophyte	On meadows, fallow lands, steppe mountain slopes
Echinops ritro L.	Perennial herbaceous	Xerophyte	On steppe meadows, gravelly and rocky slopes
E. sphaerocephalus L.	Perennial herbaceous	Mesophyte	Through mixed-grass meadows, forest edges, dry river valleys, among bushes
Filago arvensis L.	Annual herbaceous	Mesoxerophyte	In pine forests, on dry and rocky slopes of low mountains
Galatella angustissima (Tausch) Novopokr.	Perennial herbaceous	Mesoxerophyte	In the steppes, bushes, on rocky slopes
G. biflora (L.) Nees	Perennial herbaceous	Mesophyte	In the bushes, on floodplain meadows, in lighted aspen-birch groves
G. hauptii (Ledeb.) Lindl. ex DC.	Perennial herbaceous	Petrophyte	On the steppe and rocky mountain slopes
G. sedifolia subsp. *sedifolia*	Perennial herbaceous	Mesophyte	In floodplain meadows, through bushes
Hieracium krylovii Nevski ex Schljakov	Perennial herbaceous	Mesophyte	In coniferous forests, less often in birch-aspen groves
H. robustum Fr.	Perennial herbaceous	Mesoxerophyte	On the southern meadow slopes, among bushes and in rocky areas
H. umbellatum L.	Perennial herbaceous	Mesophyte	In meadows, forest edges
H. virosum Pall.	Perennial herbaceous	Mesophyte	In mixed-grass steppes, thickets of bushes, birch-aspen groves, on rocky slopes
Inula helenium L.	Perennial herbaceous	Hygromesophyte	In damp meadows, river valleys, deciduous forests.
Jacobaea erucifolia (L.) G.Gaertn., B.Mey. & Scherb.	Perennial herbaceous	Mesophyte	In aspen-birch groves, pine communities, shrub thickets, steppe slopes, and forest edges
J. vulgaris Gaertn.	Perennial herbaceous	Mesophyte	In sparse birch-aspen groves, in meadow steppes, shrubs, riverside pebbles
Jurinea albicaulis Bunge	Perennial herbaceous	Xeromesophyte	In steppe meadows, pine forests
J. cyanoides (L.) Rchb.	Perennial herbaceous	Xerophyte	Across sandy steppes, mountain slopes, and pine mountain forests
J. multiflora (L.) B. Fedtsch.	Perennial herbaceous	Xerophyte	In feather grass steppes, rocky slopes
Klasea dissecta (Ledeb.) L.Martins	Perennial herbaceous	Xeropetrophyte	In rocky and gravelly slopes, mountain steppes
Lactuca tatarica (L.) CAMey.	Annual/perennial herbaceous	Mesophyte	As a weed, in fallow lands, in crops
Leucanthemum vulgare Lam.	Perennial herbaceous	Mesophyte	In meadows, forest edges, fields, sometimes like weeds
Ligularia glauca (L.) O. Hoffm.	Perennial herbaceous	Hygromesophyte	In floodplain and dry meadows, in sparse forests
L. macrophylla (Ledeb.) DC.	Perennial herbaceous	Hygrophyte	Through wet and marshy meadows, river valleys, springs
Matricaria chamomilla L.	Annual herbaceous	Mesophyte	Near housing, along roadsides, as a weed, in crops
Omalotheca sylvatica (L.) F.W.Schultz & Sch.Bip.	Perennial herbaceous	Mesophyte	In sparse forests, bushes, forest clearings, fallow lands
Pentanema asperum (Poir.)	Perennial herbaceous	Xerophyte	In the steppe zone, steppes, steppe meadows

P. britannica (L.)	Perennial herbaceous	Mesophyte	In damp places, on forest edges, fields and vegetable gardens as a weed, on gravel
P. salicinum (L.)	Perennial herbaceous	Xeromesophyte	In steppe meadows, along river valleys, on pebbles
Picris hieracioides L.	Biennial herbaceous	Mesoxerophyte	In dry meadows, among bushes, along roads, like a weed
P. nuristanica Bornm.	Annual/perennial herbaceous	Petrophyte	Along the rocky, gravelly mountain slopes and river valleys
Pilosella echioides (Lumn.) F. W. Schultz & Sch. Bip.	Perennial herbaceous	Xerophyte	Along steppe meadows, rocky slopes, steppes, pine forests
P. echioides subsp. *echioides*	Perennial herbaceous	Mesophyte	Through the steppe meadows
Psephellus sergii (Klokov) AL Ebel	Perennial herbaceous	Xeropetrophyte	In the steppes, rocky pine forests, on rocky and gravelly slopes
P. sibiricus (L.) Wagenitz	Perennial herbaceous	Xeropetrophyte	On rocky steppe mountain slopes, in upland pine forests
Pseudopodospermum pubescens (DC.) Zaika, Sukhor. & N. Kilian	Perennial herbaceous	Xerophyte	On dry meadows, rocky and gravelly steppe slopes of low mountains, rocky areas
P. strictum (Hornem.) Zaika, Sukhor. & N. Kilian	Perennial herbaceous	Xeromesophyte	In the steppes and meadows
Rhaponticoides ruthenica (Lam.) MV Agab. & Greuter	Perennial herbaceous	Xerophyte	In the steppes, on rocky slopes, in pine forests
Rhaponticum altaicum (Fisch. ex Spreng.) Soskov	Perennial herbaceous	Xerophyte	In the steppe region on solonetzic meadows
Saussurea elata Ledeb.	Perennial herbaceous	Xeromesophyte	In steppe meadows, in thickets of steppe shrubs
S. latifolia Ledeb.	Perennial herbaceous	Mesophyte	In coniferous and deciduous forests
S. parviflora (Poir.) DC.	Perennial herbaceous	Mesophyte	In coniferous and deciduous forests, bush thickets, river valleys
S. rigida Ledeb.	Perennial herbaceous	Xeromesophyte	In the steppe zone, on meadow steppes, among shrubs, and on steppe mountain slopes
Scorzonera parviflora Jacq.	Perennial herbaceous	Hygromesophyte	In wet saline meadows
S. purpurea L.	Perennial herbaceous	Xeromesophyte	In the steppes, meadows, gravelly and grassy slopes
Scorzoneroides autumnalis (L.) Moench	Perennial herbaceous	Mesophyte	In meadows, fallow lands, near dwellings
Senecio nemorensis L.	Perennial herbaceous	Hygromesophyte	In coniferous and deciduous forests, forest meadows, swamp areas, river valleys
Serratula coronata L.	Perennial herbaceous	Mesophyte	Through forests, tall-grass forest and steppe meadows, mountain slopes
S. kirghisorum Iljin	Perennial herbaceous	Xerophyte	In the steppe zone along clay-rocky steppes
Solidago virgaurea L.	Perennial herbaceous	Mesophyte	In sparse forests, bushes, meadows and steppe clearings
Sonchus arvensis L.	Perennial herbaceous	Hygromesophyte	In damp meadows, along river banks, in thickets of bushes, in fallow lands, wastelands, vegetable gardens

S. oleraceus L.	Annual/biennial herbaceous	Mesophyte	As a weed in fields, fallow lands, vegetable gardens, near houses
S. palustris L.	Perennial herbaceous	Hygrophyte	Along the banks of rivers, streams, in damp places
Tanacetum barclayanum DC.	Perennial herbaceous	Petrophyte	Along the rocky mountain slopes
T. bipinnatum subsp. *bipinnatum*	Perennial herbaceous	Mesophyte	In fields, meadows, along river banks, as weeds near houses
T. crassipes (Stschegl.) Tzvelev	Perennial herbaceous	Xeropetrophyte	On the rocky mountain slopes, in the steppe
T. vulgare L.	Perennial herbaceous	Hygromesophyte	In forest and steppe zones, in forest clearings, along the banks and floodplains of rivers, in intermountain depressions and fallow lands
T. kittaryanum (CA Mey.) Tzvelev	Perennial herbaceous	Petrophyte	In gravelly steppes, on rocky and gravelly slopes
T. vulgare subsp. *vulgare*	Perennial herbaceous	Mesophyte	In meadows, grassy slopes, among bushes
Takhtajaniantha austriaca (Willd.) Zaika, Sukhor. & N.Kilian	Perennial herbaceous	Petrophyte	On the steppe, rocky-gravelly slopes of the foothills
Taraxacum erythrospermum Andrz. ex Besser	Perennial herbaceous	Xerophyte	In slightly saline meadows, dry rocky steppes, often as a weed
T. glabrum DC.	Perennial herbaceous	Hygromesophyte	On wet grassy slopes
T. sect. *Taraxacum* FH Wigg.	Perennial herbaceous	Mesophyte	In forest and floodplain meadows, often as a weed near housing
Tephroseris integrifolia (L.) Holub	Perennial herbaceous	Mesoxerophyte	In mixed, pine, deciduous forests, steppes, steppe meadows, rocky steppe slopes
Tragopogon alaicus S.A. Nikitin	Perennial herbaceous	Mesophyte	On mountain slopes, in pine forests, along river valleys
T. dubius Scop.	Biennial herbaceous	Mesoxerophyte	Across the steppes, dry meadows, and bushes
T. orientalis L.	Biennial herbaceous	Xeromesophyte	In meadows, mountain grassy slopes, in lighted aspen-birch groves, meadow steppes
T. pratensis L.	Biennial herbaceous	Mesophyte	Across meadows, meadow slopes, forest glades
T. ruber SG Gmel.	Annual/perennial herbaceous	Petrophyte	On clayey-gravelly and rocky slopes
T. sibiricus Ganesh.	Biennial herbaceous	Mesophyte	In meadows, forest clearings, forest edges, in lighted aspen groves
T. songoricus S. A. Nikitin	Annual/perennial herbaceous	Xeromesophyte	In the mountains, in meadows, dry slopes, edges of birch groves, among feather grass and mixed grass steppes, in bushes
Tripleurospermum inodorum (L.) Sch. Bip.	Annual/biennial herbaceous	Mesophyte	Along roads, in fields, in crops, near populated areas
Tussilago farfara L.	Perennial herbaceous	Hygrophyte	On unsodden sandy, clayey and steep banks of reservoirs
Xanthium strumarium L.	Annual herbaceous	Mesophyte	In vacant lots, roadsides, gravel areas, near houses
**Viburnaceae**			
Adoxa moschatellina L.	Perennial herbaceous	Mesophyte	In deciduous forests, shrubby shady communities

Sambucus sibirica Nakai	Bush	Mesophyte	In coniferous and mixed forests, gorges and north-eastern slopes
Viburnum opulus L.	Bush	Hygromesophyte	In woody and shrubby thickets, along the banks of rivers, springs, ravines, and gorges
**Caprifoliaceae**			
Lomelosia austroaltaica (Bobrov) Soják	Subshrub	Petrophyte	On the open south-eastern rocky slopes of the foothills
Lonicera microphylla Willd. ex Schult.	Bush	Petrophyte	Along rocky and stony slopes, in sparse rocky pine forests
L. tatarica L.	Bush	Xeromesophyte	In the steppes, shrub thickets, floodplain meadows, along river valleys, and gorge bottoms
Patrinia intermedia (Hornem.) Roem. & Schult.	Perennial herbaceous	Petrophyte	On rocky mountain slopes, cliffs, rocky steppes, in sparse pine forests
Scabiosa ochroleuca L.	Perennial herbaceous	Xerophyte	In the steppes, on steppe meadows, rocky pine forests
Valeriana rossica PA Smirn.	Perennial herbaceous	Mesophyte	In steppe meadows, forest clearings, forest edges, in bushes
V. tuberosa L.	Perennial herbaceous	Xerophyte	In gravelly steppes, in dry depressions, solonetzic steppes
**Apiaceae**			
Aegopodium alpestre Ledeb.	Perennial herbaceous	Mesophyte	On the grassy slopes
Angelica sylvestris L.	Perennial herbaceous	Hygromesophyte	In pine and deciduous forests, river and stream valleys, tall grass thickets
A. decurrens (Ledeb.) B. Fedtsch.	Perennial herbaceous	Hygrophyte	Along the banks of rivers, springs, in tall-grass meadows, in river floodplains, damp tracts
Anthriscus sylvestris (L.) Hoffm.	Biennial/perennial herbaceous	Hygromesophyte	In shady gorges, river valley forest communities
Bupleurum aureum Fisch. ex Hoffm.	Perennial herbaceous	Mesophyte	In meadows, among coniferous plantations, deciduous forests, less often, along river valleys
B. krylovianum Schischk.	Perennial herbaceous	Mesoxerophyte	In the thickets of steppe shrubs, on rocky mountain slopes
B. scorzonerifolium Willd.	Perennial herbaceous	Xerophyte	In mountain rocky steppes, steppe meadows, in pine rock plantations
Carum carvi L.	Perennial herbaceous	Mesophyte	Along streams, roads, in meadows, near housing
Chaerophyllum prescottii DC.	Perennial herbaceous	Hygromesophyte	In damp meadows, in aspen-willow groves, in gorges
Conium maculatum L.	Annual/biennial herbaceous	Mesophyte	As a weed, near housing, in landfills, along roads, in vegetable gardens, in ravines among bushes
Eryngium planum L.	Perennial herbaceous	Xeromesophyte	In the steppes, meadow depressions, and fallow lands
Ferula dissecta (Ledeb.) Ledeb.	Perennial herbaceous	Mesoxerophyte	Along the grassy steppe slopes of the foothills
F. songarica Pall. ex Willd.	Perennial herbaceous	Xeromesophyte	On the steppe grassy slopes, in the meadow steppes
Heracleum dissectum Ledeb.	Biennial/perennial herbaceous	Hygromesophyte	In damp places, in river valleys, in the belt of tree-shrub vegetation

H. sibiricum L.	Biennial/perennial herbaceous	Hygromesophyte	In thickets of bushes, damp meadows, in aspen-birch groves
Libanotis pyrenaica subsp. *pyrenaica*	Perennial herbaceous	Mesoxerophyte	In dry meadows, in meadow steppes, among bushes
Peucedanum morisonii Besser ex Schult.	Perennial herbaceous	Xeromesophyte	In mixed-grass-feather-grass steppes, thickets of shrubs, in steppe meadows
Pleurospermum uralense Hoffm.	Biennial/perennial herbaceous	Mesophyte	On the edges of the forest among the bushes
Schrenkia vaginata (Ledeb.) Fisch. & CA May.	Perennial herbaceous	Petrophyte	On the southern rocky mountain slopes
Seseli buchtormense (Fisch. ex Hornem.) W.D.J. Koch	Perennial herbaceous	Petrophyte	In rock crevices, on stony, gravelly slopes
S. incanum (Stephan ex Willd.) B. Fedtsch.	Perennial herbaceous	Petrophyte	Along the rocky slopes of the low mountains
S. ledebourii G. Don	Perennial herbaceous	Xerophyte	In mixed-grass-feather grass and fescue steppes, less often, steppe meadows
Sium sisarum L.	Perennial herbaceous	Hygrophyte	In damp places, along river banks
